# An *in vivo* CRISPR screen in chick embryos reveals a role for MLLT3 in specification of neural cells from the caudal epiblast

**DOI:** 10.1242/dev.204591

**Published:** 2025-02-12

**Authors:** Ashley R. G Libby, Tiago Rito, Arthur Radley, James Briscoe

**Affiliations:** The Francis Crick Institute, Developmental Dynamics Group, 1 Midland Rd, London, NW1 1AT, UK

**Keywords:** Neural induction, Chick, NMPs, CRISPR/cas9, Super elongation complex, *In vivo* screen

## Abstract

Tissue development relies on the coordinated differentiation of stem cells in dynamically changing environments. The formation of the vertebrate neural tube from stem cells in the caudal lateral epiblast is a well-characterized example. Despite an understanding of the signalling pathways involved, the gene regulatory mechanisms remain poorly defined. To address this, we developed a multiplexed *in vivo* CRISPR screening approach in chick embryos targeting genes expressed in the caudal epiblast and neural tube. This revealed a role for *MLLT3*, a component of the super elongation complex, in the specification of neural fate. Perturbation of *MLLT3* disrupted neural tube morphology and reduced neural fate acquisition. Mutant forms of retinoic acid receptor A lacking the *MLLT3* binding domain similarly reduced neural fate acquisition. Together, these findings validate an *in vivo* CRISPR screen strategy in chick embryos and identify a previously unreported role for *MLLT3* in caudal neural tissue specification.

## Teaser

**Summary:** A multiplexed *in vivo* CRISPR screening approach demonstrates the utility of pooled perturbation screening in chick embryos, identifying involvement of the super elongation complex component MLLT3 in caudal neural tissue specification.

## INTRODUCTION

To generate functioning tissues during development, a wide variety of cell types must be specified in a timely and organized manner. As such, individual cells transition through multiple embryonic regions (Martins-Green and Erickson, 1986; Anderson et al., 2000; Abukar et al., 2023 preprint), experience varying signalling regimes (Diez Del Corral and Storey, 2004; Yang, 2004; Zagorski et al., 2017; Van Boxtel et al., 2018; Yamaguchi, 2001), and activate distinct gene expression programmes as they differentiate towards their eventual fate (Briscoe and Ericson, 1999; Faial et al., 2015; Sauka-Spengler and Bronner-Fraser, 2008). Across tissues, developmental stages, and species, coordination of signalling processes with gene expression is necessary to ensure the robust and precise outcome of tissue development (Van Boxtel et al., 2015; Lunn et al., 2007; Hill et al., 2017).

The progressive elaboration of the vertebrate trunk from the caudal lateral epiblast (CLE) exemplifies a dynamic signalling landscape coordinating the acquisition of diverse cell fates. Here, a multipotent population of cells, termed neuromesodermal progenitors (NMPs), gives rise to successively more posterior neural and paraxial mesodermal lineages (Henrique et al., 2015; Wilson et al., 2009; Guillot et al., 2021; Tzouanacou et al., 2009) while proliferation of NMPs fuels the elongation of axial tissues (Guillot et al., 2021; Roszko et al., 2007; Sausedo et al., 1997). Consequently, the rates at which NMPs emerge, self-renew, and differentiate must be closely regulated within the CLE to balance the production of trunk tissues. To this end, the interplay between WNT, fibroblast growth factor (FGF), and retinoic acid (RA) signalling regulates the behaviours of NMPs and other caudal stem cell populations (Diez Del Corral and Storey, 2004; Henrique et al., 2015; Boulet and Capecchi, 2012; Janesick et al., 2014). Perturbations or spatial redistribution of these signals can delay or alter the acquisition of subsequent cell fates. For example, protracted FGF signalling impedes neural differentiation (Corral et al., 2002; Patel et al., 2013; Bertrand et al., 2000), RA accelerates neural fate acquisition (Wilson et al., 2004; Del Corral et al., 2003), and elevated WNT signalling drives mesodermal fates (Takada et al., 1994; Martin and Kimelman, 2012). Despite an overall understanding of the signalling molecules involved in balancing the allocation of NMP-derived trunk fates (Gouti et al., 2014; Blassberg et al., 2022), our current knowledge of the specific gene regulatory mechanisms involved in this fate decision is based on a handful of genes, leaving a gap in knowledge of the mechanisms and genes governing the rapid transitions involved in fate decisions.

In recent years, pooled CRISPR/Cas9 genetic screens targeting multiple genes have proved valuable tools for the interrogation of gene regulatory mechanisms (Dixit et al., 2016; Datlinger et al., 2017; Yao et al., 2023; Chow and Chen, 2018; Lenoir et al., 2017; Replogle et al., 2020). However, only a limited number of *in vivo* pooled screens have been performed to date, in part due to technical challenges such as the even distribution of guides, low transfection rate *in vivo*, and low cell numbers due to tissue size. As such, *in vivo* pooled screens have been restricted to more easily accessible tissues such as the brain (Chow et al., 2017; Jin et al., 2020; Ruetz et al., 2024; Zheng et al., 2024; Santinha et al., 2023), or to species such as zebrafish that are more experimentally tractable (Shah et al., 2015). Nevertheless, the outcomes of *in vivo* screens have demonstrated their potential to identify previously understudied genes involved in specific processes (Luo et al., 2022; Boehnke et al., 2022). Given the potential of *in vivo* screens, there is a need for the development of methods that allow targeting of specific tissues across multiple species.

To refine our knowledge of the molecular mechanisms that govern neural tube development, we developed a pooled *in vivo* CRISPR screen in chick embryos. Genetic targets were identified using a single-cell transcriptome dataset (Rito et al., 2025) and perturbed following electroporation of CRISPR guides into the chick CLE in a pooled fashion. Post-perturbation, enrichment or depletion of lineages arising from the CLE were assayed by single-cell RNA sequencing (scRNA-seq). Our results emphasized a multifaceted regulation of the differentiation of CLE cells to neural tube fate that involves the interaction of FGF, WNT, and RA signalling with gene regulatory programmes. Further, we identified a previously unobserved role of the gene *MLLT3*, a member of the super elongation complex (SEC), as a key factor in controlling the behaviour of cells in the CLE. While observed *MLLT3* expression was restricted to the CLE and primitive streak, targeting *MLLT3* resulted in a reduction of knockout (KO) cells both within the CLE and within the neural tube. We provide evidence that this reduction in neural fate is due to an interaction with retinoic acid receptor A (RARα). Overall, this work demonstrates a strategy for pooled perturbation screens in chick embryos, highlighting the utility of *in vivo* pooled screens to generate a mechanistic understanding of gene regulation, and provides new insight into how RA signalling is coupled with spatially defined gene expression to facilitate rapid fate acquisition and ensure the proportioned generation of tissues.

## RESULTS

### Identification of genes marking the transition from CLE to neural fate

**Fig. 1.**
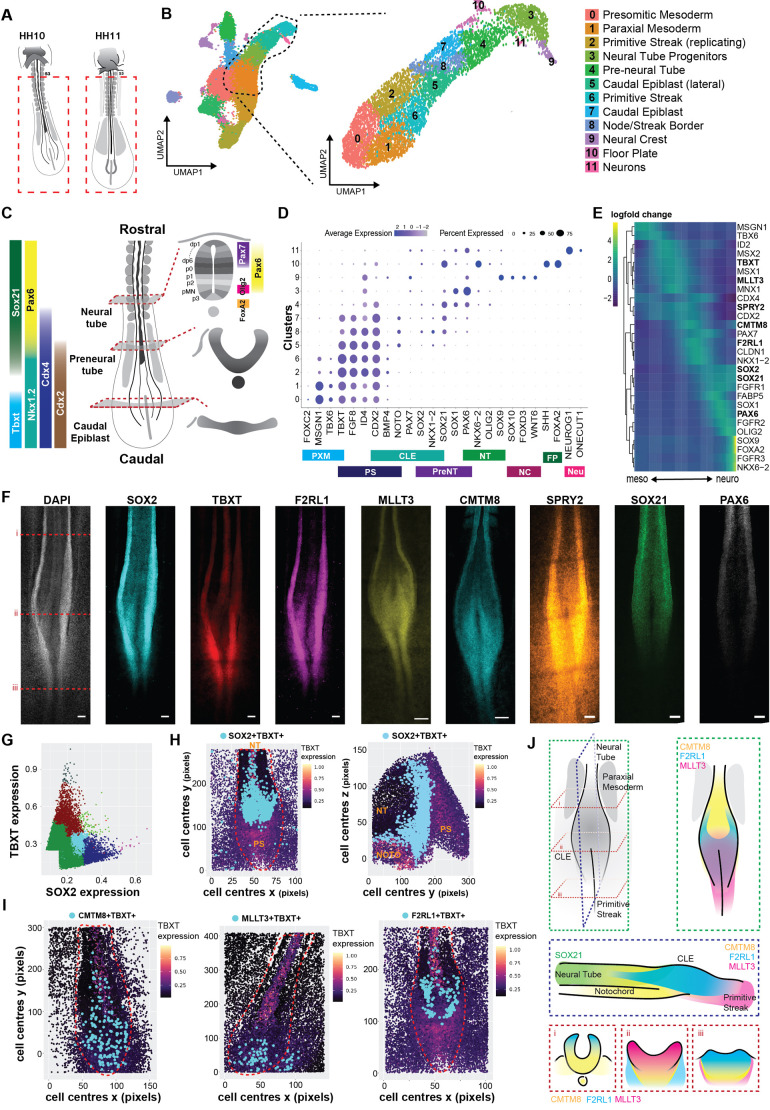
**Mapping gene expression in CLE and neural tube of chick embryos.** (A) Schematic of embryos used in a previously published dataset (Rito et al., 2025). (B) Computational selection and re-clustering of dataset focusing on the primitive streak, caudal lateral epiblast (CLE), and neural tube. (C) Adapted schematic (Joshi et al., 2019) indicating known transcription factor expression in the primitive streak, CLE and neural tube. (D) Dot plot of representative gene expression across dataset of lineage markers. (E) Heatmap of pseudo-trajectory from mesoderm to neural. Gene expression in log counts (Faial et al., 2015). (F) Example HCR images of the indicated genes in HH10 chick embryos for the data shown in E [dashed lines indicate levels of pre neural tube (i), CLE (ii), and primitive streak (iii)]. Scale bars: 50 µm. (G) Hierarchical clustering of *TBXT* transcript count with *SOX2*, *CMTM8*, or *F2RL1* transcript count (light blue dots mark the population plotted in H. (H) Left: Population of *TBXT*^+^*SOX2*^+^ cells (light blue) were mapped onto *TBXT* expression maps of the imaged embryo in the *xy* plane where rostral is top and caudal is bottom (red dashed line demarks the primitive streak, CLE, and neural tube). Right: Population of *TBXT*^+^*SOX2*^+^ cells (light blue) mapped onto *TBXT* expression maps of the imaged embryo in the *yz* plane where rostral is left and caudal is right. (I) Populations of *CMTM8*^+^*TBXT*^+^, *MLLT3*^+^*TBXT*^+^, and *F2RL1*^+^*TBXT*^+^ cells (light blue) mapped to *TBXT* expression maps of HH10 embryos where the top is rostral and bottom is caudal (red outline demarks the primitive streak, CLE, and neural tube). (J) Schematic of expression domains determined via HCR. Green boxes mark view from above (*xy*); blue box marks view from side (*yz*); and red boxes mark three cross-sectional (*xz*) views from rostral to caudal. CLE, caudal lateral epiblast; FP, floor plate; NC, neural crest; Neu, mature neurons; NOTO, notochord; NT, neural tube; PreNT, preneural tube; PS, primitive streak; PXM, paraxial mesoderm.

To investigate the control of fate transitions from the CLE to neural tube, we analysed single-cell transcriptome datasets of the chick embryo at Hamburger–Hamilton (HH) stages HH10 and HH11 (Rito et al., 2025) (Fig. 1A). We computationally isolated cells annotated as primitive streak, CLE, pre-neural tube, and neural tube based on their gene expression (Fig. 1B) and used an entropy sorting-derived feature selection algorithm (continuous entropy sort feature weighting, cESFW) (Radley et al., 2023, 2024) to identify co-regulated genes (total of 2505) in the trajectory from CLE to neural tube (Fig. S1A,B, Table S1). These genes were then used to subset the scRNA-seq data (Fig. 1B, right) and identified 12 clusters of cells (Fig. 1B,D) in which the pattern of gene expression was associated with anatomical regions of the embryo (Fig. 1C, Fig. S1D), including a population of *SOX2*, *SOX3*, and *TBXT* co-expressing cells representing presumptive NMPs (Fig. S1D, lower right). A pseudotime algorithm generated predicted lineage trajectories (Fig. 1E, Fig. S1C), providing higher resolution of cell states and highlighting several previously understudied genes. For example, expression of *MLLT3* overlapped with that of *TBXT* and *MSX1* within the primitive streak and CLE; and *F2RL1* and *CMTM8* were expressed in the CLE with *CLDN1* and *NKX1-2* (Fig. 1E).

To refine the map of *CMTM8*, *MLLT3*, and *F2RL1* expression, we examined expression of cytoskeletal components reported to mark various domains of the CLE (Rito et al., 2025): *CNTN2* (preneural), *NEFM* (node-streak border), and *ADAMTS18* (primitive streak) in the wild-type dataset (Fig. S1F). *CMTM8* largely overlapped with *NEFM* in the CLE, although the expression domain of *CMTM8* extended beyond that of the *NEFM* domain (Fig. S3F). *MLLT3* overlapped with ADAMTS18 in the primitive streak (Fig. S3F) and with NEFM in the CLE (Fig. S3F), again representing a larger domain than the cytoskeletal component. Finally, *F2RL1* had an expression domain similar to *NEFM* (Fig. S3F), largely restricted to the CLE.

To validate the observed gene overlap and spatial location of the transcriptome analysis, we performed hybridization chain reaction (HCR) in HH10 embryos (Fig. 1F). We quantified gene expression (Fig. S2) and used hierarchical clustering of cell expression profiles to identify cell populations within the CLE. Co-expression of *SOX2* and *TBXT* was identified in a population of putative NMPs within the CLE (Fig. 1G, light blue dots). These could be mapped at single-cell resolution back to the embryo [Fig. 1H, light blue dots in *xy* axis (left) and *yz* axis (right)]. To define the spatial-transcriptomic arrangement of the CLE, the expression profiles of *CMTM8*, *F2RL1*, *MLLT3*, and *SOX21* (a non-CLE expressed control) were compared to that of *TBXT* (Fig. S3A) and mapped to the embryo (Fig. 1I, Fig. S3B-D). This produced a refined CLE domain map (Fig. 1J) of *CMTM8*, *F2RL1*, and *MLLT3* expression in the CLE in which each have spatially distinct cell populations co-expressing *TBXT* (Fig. 1I, Fig. S3C-E). *CMTM8^+^TBXT^+^* cells were observed in the CLE with a minor contribution to the notochord (Fig. 1I, left, blue dots; Fig. S3C, right, blue dots). *MLLT3^+^TBXT^+^* cells were located solely in the CLE and primitive streak (Fig. 1I, centre, blue dots; Fig. S3D, left, blue dots). Cells with high levels of *F2RL1* were located sporadically outside *TBXT* regions (Fig. S2C), while medium *F2RL1* expression largely overlapped with *TBXT* in the CLE (Fig. 1I, right, blue dots; Fig. S3D, left, blue dots), and all three genes (*CMTM8*, *F2RL1*, and *MLLT3*) were expressed at low levels in the neural tube (Fig. S2C-E). Overall, this dataset highlights domain-specific patterns of gene expression in the CLE and neural tube that agree with previously reported datasets. These observations raise the question of whether these genes regulate morphogenic signal interpretation to control balanced lineage emergence.

### Adapting chick CRISPR/Cas9 system for pooled scRNA-seq screening

**Fig. 2.**
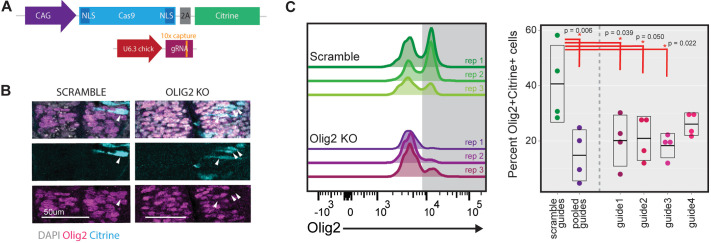
**Chick *in ovo* CRISPR system.** (A) Schematic of the two-plasmid CRISPR system, consisting of a CAG-driven CAS9-IRES-Citrine and a U6-driven gRNA with additional capture sequence for 10x feature barcoding capture. (B) Immunofluorescent images for the indicated markers in transverse sections of the neural tube 24 h post-electroporation. White arrowheads mark detection of Cas9 and in the KO condition loss of Olig2 protein. (C) Flow cytometry analysis of Olig2 in cells from dissociated embryos 24 h post neural tube electroporation (*P<0.05 by ANOVA and subsequent Tukey tests; *n*=4 embryos per condition). Horizontal lines of box plots mark the first quartile, median and third quartile distribution of data.

To investigate the function of these identified transition-associated genes expressed in the CLE, we conducted an *in vivo* pooled CRISPR screen in chick embryos. To this end, we modified a previously published, chicken-specific CRISPR system (Gandhi et al., 2017; Williams et al., 2018). The system utilizes dual plasmids; one plasmid encoded a CAG-driven Cas9 and a Citrine reporter and the second contained the chick U6.3 promoter driving a guide RNA (gRNA) in which we inserted a capture sequence in the stem loop, to allow detection in 10x scRNA-seq (Choo et al., 2021) (Fig. 2A).

To assess whether the capture sequence affected system performance, we designed four gRNAs targeting the transcription factor *OLIG2*, knock out of which in the neural tube has been well documented (Takebayashi et al., 2002; Zhou and Anderson, 2002). The gRNAs and the Cas9-encoding plasmid were introduced into HH10 chick neural tubes by neural tube injection and unilateral electroporation. The side of the neural tube that received gRNAs (right) displayed disrupted Olig2 domains compared to the non-electroporated side (Fig. S4A). Further, cells that had received the Cas9 plasmid lacked Olig2 protein (Fig. 2B, Fig. S4A). By contrast, Olig2 expression remained unperturbed in control embryos electroporated with scramble gRNAs. To quantify the efficiency, we used flow cytometry to isolate Sox2^+^ neural progenitors from the neural tubes of embryos electroporated with either the scramble gRNA or Olig2-targeting gRNA pool (Fig. S4B). This confirmed reduced Olig2 protein in electroporated cells from embryos receiving the Olig2 gRNA pool compared to the scramble control (Fig. 2C, left), suggesting that the capture sequence did not influence system performance.

With the aim of performing a pooled screen targeting multiple genes, we evaluated the efficacy of individual gRNAs in our perturbation system, as gRNA failure – failure to mutate the target gene – has been reported (Hanna and Doench, 2020; Smits et al., 2019). Flow cytometry of Sox2^+^ progenitors electroporated with individual Olig2 gRNAs revealed significantly reduced Olig2 levels from the majority of guides; however, one gRNA (guide 4) did not deplete Olig2^+^ cells significantly (Fig. 2C, right). Overall, we conclude that the changes made to the system, including the addition of the capture sequence to gRNAs, did not impair Cas9 activity. We proceeded with designing a screen in which unique barcodes paired with each guide variant allow comparison between guides as an internal quality control for guide failure.

### Preliminary test of the *in vivo* pooled screen

To determine the number of guides to use in an *in vivo* pooled screen, we conducted a pilot screen targeting the CLE in chick embryos (Fig. S5). This screen targeted the caudal epiblast using a two-plasmid system. The first plasmid contained Cas9 and mRFP-H2B driven by a minimal promoter and the *SOX2* enhancer N1 specific to the caudal epiblast (Fig. S5A) (Takemoto et al., 2006). The second plasmid contained the gRNA sequence driven by a chicken U6.3 promotor (Williams et al., 2018). We performed co-electroporation of 16 guides targeting four genes: *SOX2*, *TBXT*, *TBX6,* and *OLIG2* as well as two scramble guides in a separate set of embryos to serve as a control (Table S2), then sorted cells by fluorescence-activated cells sorting (FACS) for mRFP expression and conducted scRNA-seq (Fig. S5B).

In the initial dataset, there was a large proportion of contaminating haematopoietic lineage cells marked by LMO2, HBBA, and TAL1 (Fig. S5C), which were computationally removed from the dataset. We projected the single-cell transcriptomes onto the wild-type dataset to identify lineages of interest: primitive streak, CLE, and neural tube (Fig. S5D). The average unique molecular identifier (UMI) count of detected guides per cell was ∼2 (Fig. S5E) and overall, ∼1500/7300 cells contained captured guide sequence with about 1000 cells receiving only one guide (Fig. S5F,G, blue). This proportion increased to ∼1110/3400 cells with detectable gRNAs in the cell lineages of interest (Fig. S5F,G, orange). Across the four target genes, we found from 50 to over 600 cells containing each target specific guide (Fig. S5H). Unfortunately, neither of the scramble controls were detected within this dataset; this could be due to the low UMI detection rate or a low expression level of the scramble guide plasmid. To address these possible technical limitations, in future experiments we used the constitutive expression of Cas9 with the brighter fluorescence marker mCitrine (Fig. 2) to be able to capture and sequence an increased number of cells and therefore increase the likelihood of guide detection. Additionally, the scramble guides were added to the pool of KO guides instead of being run in separate embryos.

Despite scramble drop out, we were able to use the pilot dataset and the previous HCR images (Fig. 1F) to estimate how many guides could be electroporated simultaneously and thus how many target genes we could assay (Fig. S5I,J). Using the overlap of *SOX2* and *TBXT* expression, we counted 7487 cells in the CLE region targeted by electroporation. Accounting for inter-embryo variability, we estimated that the target tissue would range from 6000 to 9000 cells, allowing for 20-30 guides to be electroporated into one embryo (Fig. S5I,J). Overall, this highlights how the limited tissue size places constraints on *in vivo* screens. As such, we sought to utilize molecular insights from our re-analysis of the published transcriptome dataset (Fig. 1) to investigate neural tube fate acquisition from axial progenitors. Further, we pooled multiple embryos to increase the number of guides to target 25 genes. As a result, we generated a pooled gRNA library to target 25 candidate genes. In total, the library consisted of 100 gRNAs (four gRNAs per gene) and two additional scramble controls for a total of 102 guides (Table S2).

### Generating an *in vivo* pooled screen for regulators of neural differentiation

To generate a list of candidate genes involved in the CLE-to-neural transition, cESFW analysis was used to generate ranked gene lists for each cluster in the mesoderm to neural lineage trajectory (Table S1). The entropy sort score (ESS) correlation metric was used to identify the top 250 genes specifically enriched in a cluster. From this, we refined a list of 25 target genes. This list included signalling receptors and pathway components from key pathways at play (*FGFR1*, *FGFR2*, *FGFR3*, *DUSP6*, *KRAS*, *SPRY2*, *RARA*, *RARB*), several reference genes (*TBXT*, *PAX7*, *OLIG2*), the KOs of which have well-studied phenotypes (Takebayashi et al., 2002; Zhou and Anderson, 2002; Wilkinson et al., 1990; Wilson et al., 1993; Pennimpede et al., 2012; Mansouri et al., 1996; Mansouri and Gruss, 1998), and CLE transition genes identified by cESFW (*CLDN1*, *EMX2*, *FABP5*, *GREB1*, *MNX1*, *MSX1*, *MSX2*, and *NRIP1*). Of the transition genes, several have been reported to be involved in axial elongation [*GREB1* (Prajapati et al., 2020), *MNX1* (Han et al., 2020), *MSX1* and *MSX2* (Foerst-Potts and Sadler, 1997)], while others are associated with neural development [*FABP5* (Sharifi et al., 2013) and *EMX2* (Cecchi, 2002)]. As FGF and RA pathway receptors and effectors were identified by the cESFW, we also included *RARG* (retinoic acid receptor gamma-like; *LOC107051537*), *MAPK3* (ERK1; effector in the FGF pathway), and *YAP1* (reported to regulate primitive streak dynamics; Rito et al., 2025; Hsu et al., 2018). Further, we additionally included the three genes *F2RL1*, *CMTM8*, and *MLLT3*, which showed distinct expression patterns within the CLE, all of which ranked in the top 150 ESS scored genes.

**Fig. 3.**
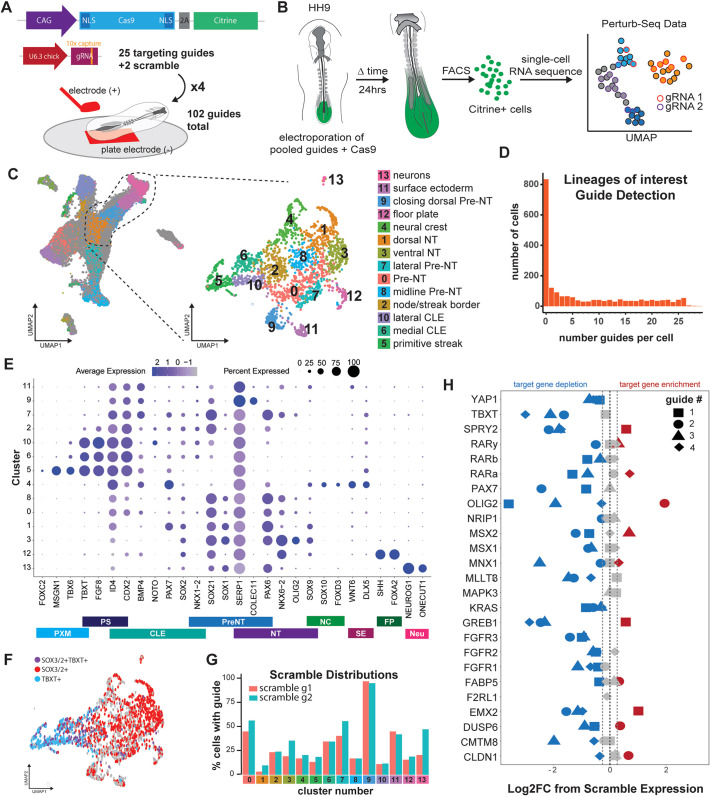
**Pooled *in vivo* screen in chick embryos.** (A) Plasmid system used to generate CRISPR KO with 25 targets in which four replicates of ten embryos where each embryo received 25 guides were pooled to generate 100 targeting guides with two additional scramble guides (102 guides total). (B) Schematic of electroporation and screening workflow. HH9 chicken embryos were electroporated with CRISPR plasmids *ex ovo*, allowed to develop for 24 h and subsequently enriched for electroporation via FACS of Citrine-positive cells. Cells were then profiled by scRNA-seq. (C) UMAP projection of scRNA-seq data into a wild-type reference map (left) and subsequent selection and re-clustering of primitive streak, CLE and neural tube (right). (D) Number of unique guide barcodes detected per cell across the lineages of interest after filtering for ambient RNA amplification. (E) Dot plot representing gene expression across the screen dataset of lineage markers. (F) UMAP showing NMPs in screen dataset marked by co-expression of *SOX2/3* and *TBXT*. (G) Percentage of cells that display a scramble gRNA barcode distributed across clusters of screen dataset. (H) Log fold change of differential expression in genes targeted by each guide in cells receiving the indicated gRNAs compared with those that received scramble [blue: reduction in expression compared to scramble containing cells; red: increase compared to scramble containing cells; grey: no change in expression; dashed lines mark 5% false discovery rate (FDR)]. CLE, caudal lateral epiblast; FP, floor plate; NC, neural crest; Neu, maturing neurons; NT, neural tube; PreNT, preneural tube; PS, primitive streak; PXM, paraxial mesoderm.

We then performed targeted electroporation of the plasmid pool to the CLE of HH9 chick embryos (Fig. 3A; *n*=4 replicates of a 25 guide mix with ten embryos/replicate) and tracked the outcome of individual gRNAs by examining the fates of cells 24 h post-electroporation by enriching Cas9^+^ cells by FACS and scRNA-seq (Fig. 3B). We hypothesized that a knockout could result in cell death (loss of guide detection), increased cell proliferation (increased guide detection), changes in cell identity (increase or loss of guide detection in particular fates), or misregulated gene expression within a cell.

We projected the single-cell transcriptomes of targeted cells onto the wild-type dataset, subsetted cells classified as primitive streak, CLE, and neural tube, and then re-clustered (Fig. 3C). From this, 14 clusters were annotated using the same markers as the wild-type dataset (Fig. 3E, Fig. S4A). Similar to the wild-type dataset, we identified *SOX2*-, *SOX3*- and *TBXT*-expressing NMPs, marking the CLE (Fig. 3F). We additionally observed a cluster of *NEUROG1*^+^ neurons, a cluster that expressed *WNT6*-, *DLX5*- and *SERP1*-positive surface ectoderm, and *BMP4^+^COLEC11^+^* cells, which is consistent with closing of the dorsal neural tube (Fig. 3E,F, Fig. S4A).

To determine KO phenotypes, we used gRNA capture sequences to identify perturbations present in individual cells. Following stringent filtering steps excluding amplification below two standard deviations of the mean as ambient RNA amplification (Fig. S6B), we found 0-29 detectable guides per cell (Fig. 3C); 1203 cells contained guides with an average of 380.27 cells for each guide sequence (Fig. 3D, Fig. S6C,D). Importantly, both scramble gRNAs were detected in the dataset and of the 102 guides used only five were undetected in the final dataset: PAX7 guide 4, MAPK3 guide 4, and F2RL1 guides 1-3.

It has been previously reported that individual cells receiving multiple guides may have confounding effects of multiple KOs. However, trends for a particular perturbation would be maintained across all cells that had received that guide (Yao et al., 2023). To test this, the distribution of cells with either scramble guide regardless of multiple KOs was compared under the assumption that the distribution of the two scramble guides would resemble one another. Despite some heterogeneity in guide distribution across clusters (Fig. 3G), the two scramble guides were equally represented (by ANOVA and subsequent Tukey tests), consistent with previously published results of pooled screens *in vitro* where multiple guides per cell were detected. We therefore used the average of the two scrambles to represent expected representation of gRNA in each of the clusters.

As one of the *OLIG2* guides did not effectively deplete Olig2 (Fig. 2C), we assumed guides targeting other genes would have a similar trend. Thus, we defined ‘successful’ guides as those which reduced target gene expression in cells that received a target gRNA compared to those that received a scramble gRNA (Fig. 3H). This stringent criterion yielded two or three ‘successful’ guides per gene on average. Notably, the sole detected *F2RL1* guide (guide 4) was ‘unsuccessful’, which, in addition to the dropout of F2RL1 gRNAs 1-3, suggests that this gene may be necessary for cell survival in the CLE. While possible dropout could be a result of lower concentration of *F2RL1* guide, the hypothesized cell death with *F2RL1* KO supports previous evidence that in mouse the loss of *F2RL1* is partially embryonic lethal (Damiano et al., 1999) and loss of both *F2RL1* (also known as proteinase-activated receptor 2, *PAR-2*) and *F2R* (*PAR-1*) simultaneously results in neural tube defects (Camerer et al., 2010) in mouse. Overall, we were able to develop and confirm the successful targeting of multiple genes in a pooled manner within single embryos.

### Guide enrichment highlights importance of FGF and RA signalling in epiblast to neural fate transition

**Fig. 4.**
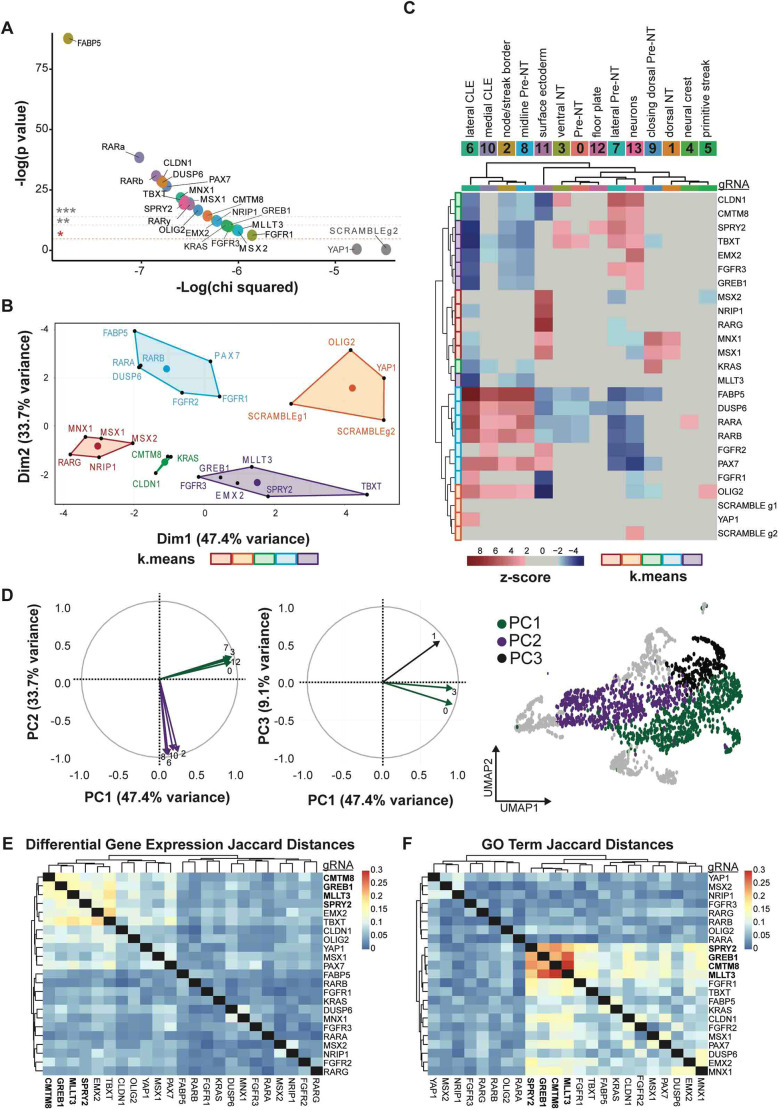
**Genes affecting transition from epiblast to neural tube.** (A) χ^2^ tests comparing distribution of gRNA-containing cells to the average across all gRNAs and to the distribution of scramble 1. Non-significant gRNAs are labelled in grey. (*P<0.05, **P<0.001, ***P<0.0001). (B) K-means clustering of gRNA enrichment scores for each gRNA. Cluster number was determined by elbow plot of the intra-cluster distances. (C) Heatmap of gRNA-containing cell enrichment compared to scramble within each cluster of the dataset, marked by χ^2^ test *z*-scores above 2 and below −2. (D) Left: Radial plots of principal component analysis of enrichment between KOs depicting which clusters are responsible for each component. Right: Schematic of screen dataset UMAP coloured by drivers of each principal component (PC1, purple; PC2, green; PC3, black). (E) Heatmap of calculated Jaccard distances between each set of identified differentially regulated genes post-knockout. (F) Heatmap of Jaccard distances based on the list of GO terms generated from the differentially regulated genes of each KO.

As cells in the CLE generate multiple cell types, we examined whether specific gRNAs altered the distribution of cell types by assessing guide enrichment compared to scramble controls. First, we used χ^2^ tests to compare guide representation in each cluster to both the scramble guide 1 and the average of all guides (Fig. 4A). Only *YAP1* gRNA-containing cells did not differ from scramble controls, indicating that *YAP1* perturbation had no observed effect on lineage fate proportions. However, χ^2^ tests are sensitive to populations with low numbers, we sought to confirm the χ^2^ results using a Kullback–Leibler divergence (KLD) test, comparing the distribution of targeting guides across clusters to the average scramble distribution (Fig. S7A). Of the 25 gene KOs analysed (excluding *F2RL1* and *MAPK3* with no successful guides from previous filtering steps), only *YAP1* KO did not significantly differ from scramble, corroborating the χ^2^ analysis.

We then calculated gRNA enrichment or depletion relative to scramble for each cluster (Fig. 4C). K-means clustering segregated gRNA containing cells into five classes based on enrichment/depletion patterns (Fig. 4B,C). The two scramble gRNAs and *YAP1* gRNAs clustered together. The *OLIG2* gRNA enrichment pattern also clustered with the scramble and *YAP1* gRNAs; this might reflect the earlier stage of development and the limited number of neural progenitors in the screen dataset compared to the pilot experiments. Principal component analysis revealed the largest variation in enrichment/depletion (47.4%) was driven by depletion in clusters representing ventral neural tube progenitors and pre-neural tube (Dim1; Fig. 4D). PC2 (33.7% variation) represented enrichment/depletion in clusters corresponding to the CLE, node-streak border, and medial pre-neural tube (Dim2; Fig. 4D). PC3 (9.1% variance) involved clusters representing the dorsal neural tube (Fig. 4D). This cluster analysis reveals that many perturbations either affected the ability of cells to become neural (PCA1) or their ability to remain in the CLE and maintain stemness.

Following the overarching trend of tight regulation of CLE exit, gRNAs targeting genes encoding FGF receptors 1 and 2, the ERK/FGF inhibitor DUSP6, and retinoic acid receptors RARα and RARβ clustered together and showed enrichment of cells in clusters containing axial progenitors and depletion in those containing neural fates. FGF and retinoic acid pathways regulate the establishment of the neuroepithelium and the exit of cells from the CLE with opposing roles in maintaining stemness (FGF) and promoting differentiation (RA) (Diez Del Corral and Storey, 2004; Del Corral et al., 2003). The co-enrichment of FGF receptor and RAR KOs in the CLE is consistent with the necessity of these pathways for the CLE-to-neural transition. Additionally, *FGFR3* and the FGF inhibitor *SPRY2* were depleted from the CLE, clustering with steroid signalling or WNT signalling (*GREB1*, *TBXT*, *MLLT3* and *EMX2*). This suggests roles for these pathways in maintaining CLE populations. Overall, examining changes in KO cell ratios identified two main contributors to acquisition of cell fate: the ability to differentiate to neural fates and an increase/depletion from the progenitor pool of the caudal epiblast.

### *CMTM8*, *GREB1*, *MLLT3*, and *SPRY2* perturbation causes similar downstream gene misregulation

As the perturbation of transition-associated genes affected the CLE-to-neural fate commitment, we then investigated disrupted gene expression that might lead to this change in differentiation. To test this, we first examined the full set of gRNAs, regardless of enrichment/depletion distributions compared to scramble controls. To focus on neural fate decisions, we analysed changes in the expression of the top 500 cESFW-selected genes per cluster from our wild-type dataset (2505 total genes). Using the package scMAGeCK, designed to identify gene regulation in pooled CRISPR screens (Yang et al., 2020), we generated lists of differentially expressed genes resulting from each gRNA and performed K-means clustering to group KOs with similar effects. There was a high level of variability between the differentially expressed genes for each KO, exemplified by the near-linear intra-cluster distance loss (Fig. S7C). However, calculating the Jaccard distances between the misregulated genes for each gRNA (Fig. 4E) revealed a group of consistently misregulated genes associated with *CMTM8*, *GREB1*, *MLLT3*, *SPRY2*, *EMX2*, and *TBXT* gRNAs (maximum Jaccard distance of 0.2, indicating 20% overlap).

As the screen also highlighted the regulation of WNT, FGF, and RA signalling pathways, we investigated whether the same biological pathways and processes were affected with different gRNAs. Examining Gene Ontology (GO) and KEGG pathway categories for the biological processes that were affected by the differentially expressed genes of each gRNA, revealed 30-40% overlap (Fig. 4F, Fig. S7B). The GO term clustering highlighted the importance of the FGF and RA signalling pathways in establishing trunk lineages. Jaccard distances of KOs associated with RA and FGF/ERK hierarchically clustered separately (Fig. 4F, left and right, respectively). These two signalling pathways have been repeatedly implicated in generating the diversity of cell types within the spinal cord and neighbouring somites (Del Corral et al., 2003; Martínez-Morales et al., 2011; Sasai et al., 2014) and our screen results corroborate these findings. Moreover, perturbations to the previously highlighted group of gRNAs (*SPRY2*, *GREB1*, *CMTM8*, and *MLLT3*) displayed the highest GO term and KEGG pathway overlap (20-30%). This is consistent with the role of *SPRY2* in fine-tuning FGF signalling in the trunk (Olivera-Martinez et al., 2012) and loss of *GREB1* affecting downstream WNT signalling and trunk elongation (Prajapati et al., 2020). However, the roles of *CMTM8* and *MLLT3* in the regulation of trunk development have yet to be explored.

### *MLLT3* gRNAs affect multiple trunk signalling axes

**Fig. 5.**
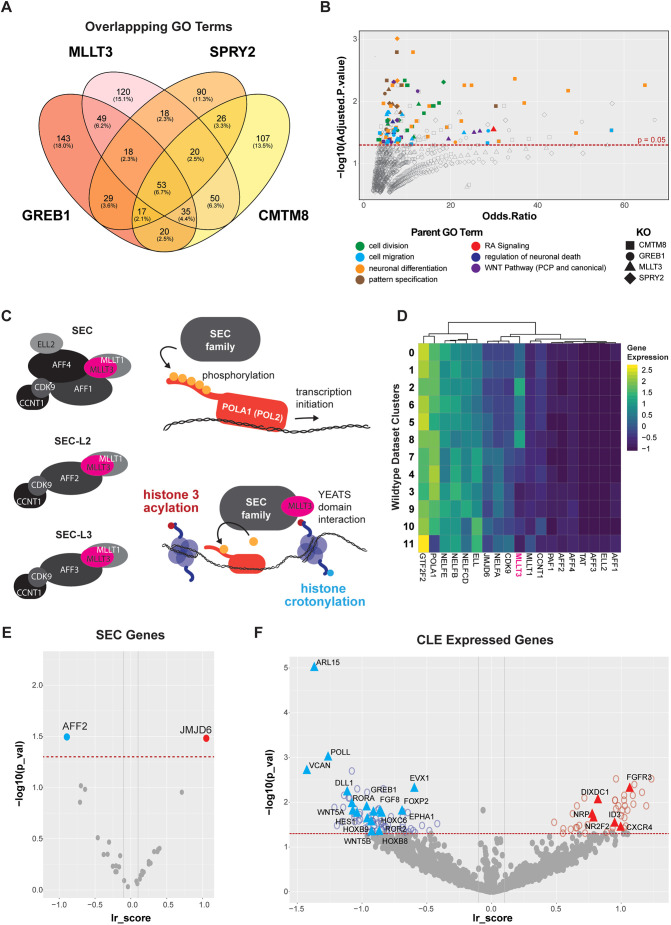
**Targeting MLLT3 disrupts Wnt PCP and RA signalling in the CLE.** (A) Venn diagram showing the overlapping percentages of GO terms between *MLLT3*, *SPRY2*, *GREB1*, and *CMTM8*. (B) Significance of GO terms with each colour marking the parent classification of GO terms (green, cell division; light blue, cell migration; yellow, neuronal differentiation; brown, pattern specification; red, retinoic acid signalling; navy blue, regulation of neuronal cell death; purple, Wnt signalling) and shape indicating the KO. Red dashed line indicates adjusted *P*=0.05 by Fisher's exact test. (C) Schematic of mammalian super elongation complex family members (SEC, SEC-L2, SEC-L3) and the phosphorylation of Poll (*POLA1* in chicken). (D) Heat map indicating the detected members of the SEC family and related complexes in the wild-type reference scRNA-seq dataset. *MLLT3* is highlighted in pink. (E) Differential regulation of SEC family members. (F) Differentially regulated genes post *MLLT3* perturbation, from the ESS ranked gene list. In E,F, lr score is a corrected read out for differential gene expression (Yang et al., 2020) and red line indicates *P*=0.05 FDR.

From our gRNA enrichment analysis, *SPRY2*, *GREB1*, *CMTM8*, and *MLLT3* gRNAs were depleted in the CLE. To investigate this further, we examined overlapping misregulated genes between the perturbations (Fig. S8A). All four gRNAs affected WNT pathway genes (*WNT5A*, *TBXT*), decreased *FGF8*, and increased *FGFR3* expression, highlighting a potential role in self-renewal of progenitors within cells of the CLE. Examining the parent GO terms between gRNAs (Fig. 5A) indicated processes such as cell division, migration, neuronal differentiation, pattern specification, retinoic acid signalling, regulation of neuronal death, and WNT signalling (Fig. 5B). *MLLT3* perturbation alone was associated with both WNT signalling and RA signalling, connecting *MLLT3* to two pathways regulating stemness in the CLE (WNT) and neural differentiation (RA). We therefore focused on *MLLT3* to elucidate its role within the CLE and neural tube.

*MLLT3* is a YEATS family member and SEC component (Pennimpede et al., 2012; Mansouri et al., 1996; Mansouri and Gruss, 1998; Prajapati et al., 2020). It participates in the three mammalian forms of SEC (Fig. 5C) to facilitate rapid gene induction by interacting with acetylated/crotonylated histone lysines (Zhao et al., 2017; Li et al., 2014, 2016; Luo et al., 2012a). *MLLT3* has primarily been studied in cancer, where its truncation and fusion with *MLL1* leads to aberrant H3K79 methylation, dysregulating the JNK (Sun et al., 2017) and WNT (Zhang et al., 2019) pathways. In developmental contexts, *MLLT3* has been implicated in vertebrae malformations (Collins et al., 2002) haematopoietic stem cell self-renewal (Calvanese et al., 2019), and haematopoietic lineage decisions (Pina et al., 2008). MLLT3 has also been shown to bind RARα and activate neurogenic programmes *in vitro* in mammalian cells (Flajollet et al., 2013), revealing a possible role in regulating a range of signalling pathways.

Because little is known about the SEC complex members in chicken, we first examined the reference wild-type scRNA-seq dataset to determine whether the components of mammalian SEC complexes were expressed (Fig. 5D). We found that components of SEC-L2 and SEC-L3 were expressed in the epiblast and neural tube indicating possible transcriptional regulation by all three complexes in chicken, similar to mammals. Of the expressed components, only *MLLT3* was differentially expressed across the CLE to neural lineage trajectory and as such was the only SEC component that was selected by the previous entropy sorting analysis as a hit for lineage regulation. Further, of the proteins associated with the SEC, the demethylase *JMJD6* (Chang et al., 2007) and the scaffold protein *AFF2* were up- and downregulated, respectively, with *MLLT3* perturbation (Fig. 5E), indicating a loss or change in composition of the SEC complex formation in the absence of *MLLT3*.

To investigate the effect of *MLLT3* perturbation on gene expression, we examined differentially expressed genes in the *MLLT3* gRNA-containing cells compared to scramble (Fig. 5F). Genes associated with RA signalling and neural differentiation, such as *RORA*, *EVX1*, and *FOXP2* (Wilson et al., 2004; Giguere et al., 1994; Pierani et al., 1999), were reduced in *MLLT3*-perturbed cells, while genes repressed by RA signalling, such as *NR2F2* (Love and Prince, 2012; Park et al., 2003), were upregulated. Additionally, WNT PCP pathway genes important for caudal epiblast fate (*WNT5A*, *WNT5B*, *ROR2*) (Guo and Xing, 2022) and Notch signalling genes involved in neural fate acquisition (*DLL1*, *HES1*) were downregulated. The misregulation of multiple pathways with opposing functions positions MLLT3 as a possible connection between CLE stem cell maintenance and acquisition of neural fate. This prompted us to investigate further the role of *MLLT3* in epiblast and neural tube lineage transitions.

### MLLT3 regulates neural tube fate acquisition through retinoic acid receptor binding

**Fig. 6.**
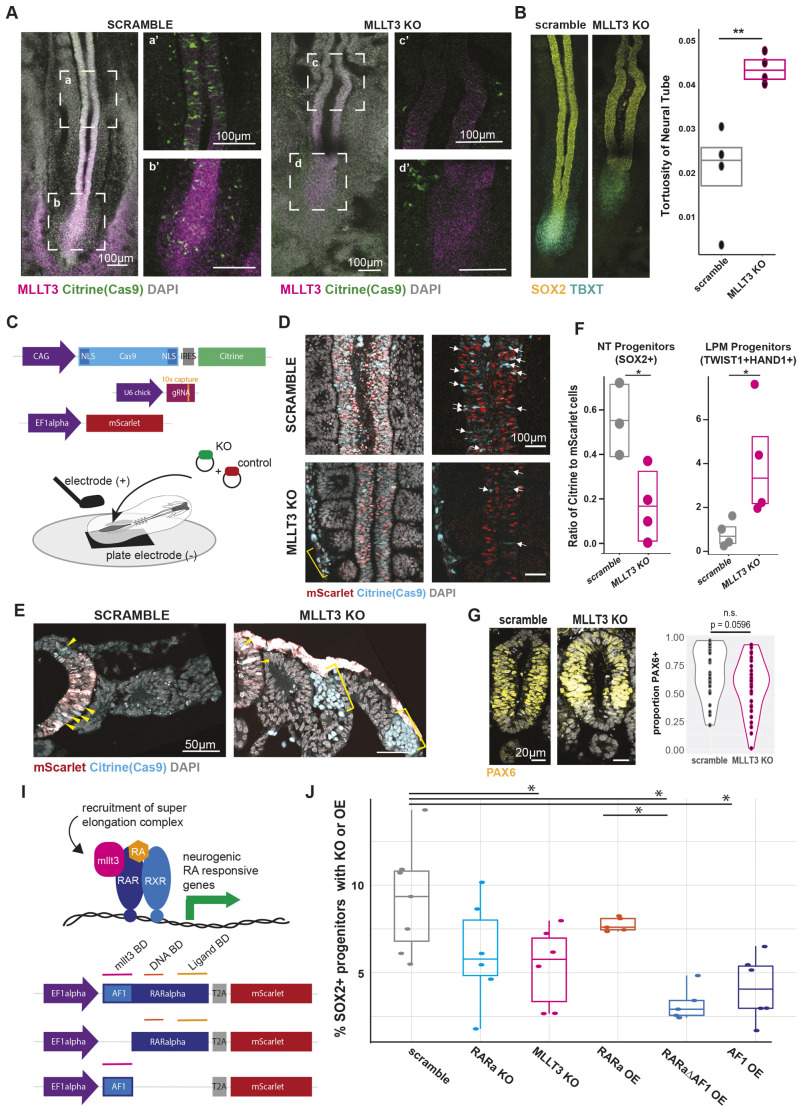
**MLLT3 regulates neural progenitor formation through RARα interaction.** (A) Fluorescence images of hybridization chain reaction for *SOX2*, *TBXT*, *MLLT3*, and reporter fluorescence of Citrine (marking Cas9-containing cells) for scramble (left) and *MLLT3* (right) gRNA-transfected embryos. (B) Left: Paired HCR images from A depicting *SOX2* and *TBXT* expression in embryos 24 h post-perturbation. Right: Quantification of changes in tortuosity (perimeter/area) of neural tube based on masks of *SOX2* expression in embryos 24 h post-perturbation. *n*=4 embryos per condition. **P<0.01 by unpaired, two-tailed *t*-test. (C) Schematic of two-colour electroporation of caudal epiblast of HH8 embryos. (D) Whole-mount immunofluorescence images showing loss of Citrine (Cas9) in *MLLT3* KO neural tubes and maintenance of mScarlet electroporation control. Yellow bracket marks MLLT3 KO cells outside of the neural tube. (E) Serial sections of embryos electroporated with either scramble or *MLLT3* gRNA and mScarlet. Yellow arrowheads mark scramble or KO cells in the neural tube. Yellow brackets show MLLT3 KO cells marked by Cas9 expression in the lateral plate mesoderm. (F) Flow cytometry of SOX2^+^ neural progenitors (left) or TWIST1^+^HAND1^+^ lateral plate mesoderm (right) in scramble versus *MLLT3* KO embryos where the *y*-axis indicates the ratio of mScarlet to Citrine-positive cells (*P<0.05 by unpaired, two-tailed *t*-test; *n*=4-5 embryos per condition. (G) Left: Immunofluorescence sections of MLLT3 KO embryos or scramble control stained for Pax6 protein. Right: Quantification of Pax6^+^ cell proportion normalized to number of cells in neural tube (six embryos per condition with ten quantified sections) n.s., not significant by unpaired, two-tailed *t*-test). (H) Top: Schematic of the possible action of Mllt3 to regulate the RA signalling response. Bottom: Schematic of constructs of RARα indicating the Mllt3-binding domain (Mllt3 BD), the DNA-binding domain (DNA BD) and the ligand-binding domain (ligand BD), visualized by mScarlet. (I) Quantification of flow cytometry analysis of SOX2^+^ cells in embryos that have been electroporated with the indicated CRISPR construct or overexpression constructs. *P<0.05 by ANOVA and subsequent Tukey tests. *n*=5-7 embryos per condition. Horizontal lines of box plots represent the first quartile, median, and third quartile of the data and whiskers mark the maximum and minimum of the dataset.

To investigate the role of *MLLT3* without the potential confounding factors of a pooled screen, we repeated individual *MLLT3* targeting the CLE of HH9 chick embryos. Consistent with the screen results, after 24 h MLLT3 gRNAs caused both a reduction in *MLLT3* expression (Fig. 6A, Fig. S9A-D) as well as a disruption of the *MLLT3* expression domain in the tail bud (Fig. S9E). Further, MLLT3 KO embryos displayed kinked spinal cords with higher tortuosity (Fig. 6B). Additionally, we observed a reduction of *MLLT3* gRNA containing cells in the neural tube and tail bud (Fig. 6Aa′-Ad′). To test whether this reduction was cell-autonomous, we co-electroporated the *MLLT3* CRISPR targeting constructs with a non-targeting Ef1a:NLS-mScarlet construct (Fig. 6C). Here, the mScarlet cells serve as a within-embryo control that should maintain their ability to differentiate to neural tube lineages. Whole-mount staining, serial sections, and flow cytometry revealed depletion of Citrine^+^ cells from the neural tube in the *MLLT3* gRNA transfected condition, while control mScarlet^+^ cells in the same embryo were present in the neural tube and maintained Sox2 expression (Fig. 6D-F, Fig. S10B,C). Importantly, the total Citrine:mScarlet ratio of cells remained consistent between scramble and *MLLT3* gRNA conditions (Fig. S10D), indicating that *MLLT3* disruption does not cause gross cell death, but rather affects cell fate and results in depletion from neural tube lineages.

Further, *MLLT3* KO increased the proportion of mScarlet cells in the lateral plate mesoderm, marked by co-expression of *TWIST1* and *HAND1* (Fig. 6E,F, Fig. S10E), suggesting that a switch in fate occurred after *MLLT3* depletion. Because of the morphological and cell fate changes after *MLLT3* KO, we examined whether KO embryos maintained neural progenitor fates (Fig. 6G). There was no significant change in PAX6 expression, which marks neural progenitors, between scramble and *MLLT3* KO embryos.

As *MLLT3* interacts with RARα and contributes to neural differentiation *in vitro* (Flajollet et al., 2013) (Fig. 6H), we investigated whether the depletion of *MLLT3* KO cells in the neural tube is due to RARα binding and presumed elongation complex recruitment to neural genes. We compared caudal epiblast-specific *MLLT3* perturbation to the forced expression of mutant RARα proteins: one lacking the MLLT3-binding domain (ΔAF1), and one of the AF1 binding domain alone (Fig. 6H). Flow cytometry analysis of SOX2-expressing neural tube progenitors confirmed a decrease in neural progenitors compared to scramble when targeting *MLLT3* (Fig. 6I). Depletion by CRISPR gRNA of RARα or overexpression of wild-type RARα had no significant effect, potentially due to compensatory mechanisms. However, forced expression of both RARα mutant constructs phenocopied *MLLT3* KO with reduced SOX2^+^ neural progenitors (Fig. 6I, right). Together, this provides evidence of a previously undocumented role of *MLLT3* in the epiblast-to-neural transition via interaction with the retinoic acid pathway and downstream genes.

## DISCUSSION

The caudal epiblast of vertebrate embryos contains populations of cells that generate the tissues of the trunk during axis elongation. The coordinated generation of these tissues depends on the signalling dynamics to determine the rate at which cells differentiate and the proportions that are allocated to specific lineages. How this is organized, and the gene regulatory mechanisms responsible for the process remain poorly defined. Mapping gene expression in the CLE called attention to several factors that might have functional roles in the elaboration of trunk tissues. To test this, we establish methods for multiplexed *in vivo* CRISPR screening in chick embryos by incorporating capture sequences to gRNAs in a chick-specific CRISPR system (Gandhi et al., 2017; Williams et al., 2018). The screen successfully enriched for regulators of cell fate decisions, evidenced by the clustering of cells containing gRNAs targeting specific signalling pathways and gene expression programmes associated with neural fate specification. Further, the screen identified a previously undocumented role for *MLLT3*, a component of the SEC, in the acquisition of neural cell fate. This highlights the power of combining transcriptomics with functional genomics. More generally, the validation of the CRISPR system for functional *in vivo* assays, paired with the historically well-characterized embryology of the chick and the potential for regionally targeted electroporation (Nakamura and Funahashi, 2001; Voiculescu et al., 2008), opens up many possibilities for interrogating gene regulatory mechanisms *in vivo* in a highly tractable system.

The screen highlighted the importance of RA, WNT, and FGF signalling pathways in regulating the epiblast-to-neural transition. Perturbation of FGF receptors, the ERK inhibitor DUSP6, and retinoic acid receptors resulted in the accumulation of cells in the caudal epiblast and depletion of ventral neural fates (Fig. 4C), consistent with the roles of FGF and RA in self-renewal and differentiation, respectively. FGF signalling is needed to trigger early neural differentiation (Bertrand et al., 2000), emphasizing the need for precise regulation of signalling. Conversely, targeting the FGF inhibitor gene *SPRY2* and WNT pathway components depleted the epiblast progenitor pool, suggesting these genes are essential for CLE maintenance. These results confirm and extend our understanding of the dynamic interplay between these signalling cascades in controlling fate decisions in the CLE.

Further, the screen identified a previously unobserved role for the gene MLLT3 as a key regulator for the initiation of the neural gene expression programme. An epigenetic reader of histone lysine acetylation and crotonylation, MLLT3 contributes to gene expression as part of the SEC (Zhao et al., 2017; Luo et al., 2012a,b). In development, MLLT3 has been studied in mammalian haematopoiesis (Mckinney-Freeman et al., 2012), and further maintains stemness in adult haematopoiesis and its overexpression expands and increases engraftment of transplanted haematopoietic stem cells (Calvanese et al., 2019). Our analysis indicated that loss of MLLT3 disrupted caudal epiblast fate maintenance (Fig. 3C), possibly prematurely depleting this stem cell reservoir and compromising neural tube formation (Fig. 6). Transcriptomic analysis of MLLT3 KO cells suggested dysregulation of multiple signalling pathway components implicated in neural induction, including retinoic acid, WNT, and Notch signalling cascades. The specific factors misregulated, such as decreased expression of RA-induced neurogenic genes (*RORA*, *ROR2*, *EVX1*, *FOXP2*) and genes encoding components of the WNT/PCP and Notch pathways (WNT5A/B, DLL1, HES1), provide a possible mechanistic explanation for the impaired neural fate acquisition upon MLLT3 perturbation.

MLLT3 has been shown to interact with a variety of transcription factors to influence differentiation programmes. For example, binding to GATA1 directs erythroid and megakaryocyte differentiation (Pina et al., 2008), and interacting with TET2 drives cortical neuron generation (Qiao et al., 2015). Direct binding of MLLT3 to retinoic acid receptors recruits the SEC to rapidly induce neural programmes (Flajollet et al., 2013). Moreover, a mutant form of RXRα disrupts proliferation in murine KMT2A-MLLT3 leukaemia cells (Di Martino et al., 2022). Consistent with a relationship between MLLT3 and RA signalling, our results from complementary approaches indicate that disrupting the ability of RARα to interact with MLLT3 phenocopies the neural tube defects observed with perturbation of *MLLT3*. By facilitating RARα-dependent activation of neural genes, MLLT3 helps establish the precise balance of progenitor differentiation and cell fate commitment required for proper neural development. This suggests that transcriptional regulation via MLLT3 and the SEC may be a conserved strategy across tissues for controlling cell fate transitions. This mechanism exemplifies how cells can achieve rapid, coordinated transcriptional responses to inductive signals by regulating RNA polymerase II elongation. The ability of the SEC to drive distinct differentiation programmes in different lineages indicates it serves as a general mechanism for converting external signals into cell type-specific transcriptional outputs. This establishes regulation of RNA polymerase II pause-release as a fundamental control point for coordinating cell fate specification during development. Examining the interplay between signal-responsive transcription factors and elongation complexes will be crucial for understanding fate specification across contexts.

While this focused approach successfully identified MLLT3, expanding the size of our gRNA library could uncover additional regulators and provide a more comprehensive view of the epiblast-to-neural transition. The pooled screening approach results in individual cells receiving multiple guides targeting different genes. Although this did not appear to affect the average distributions of KO cells in our study, and has been shown previously to be an effective screening method (Yao et al., 2023), it may mask subtle effects on gene networks or lineage commitment. Furthermore, considering only those guides that decreased the level of the targeted transcript may unnecessarily exclude some data from the analysis. Although we demonstrate loss of neural progenitors with mutant RARα constructs directed at the AF1-binding domain, this domain interacts with co-factors other than MLLT3 and disruption of these interactions might contribute to the observed effects. For example, BRD4 interacts with the AF1 domain (Flajollet et al., 2013). Although expression of BRD4 was not detected in either the wild-type or the screen datasets, it indicates the possible action of other co-regulators in this process.

In summary, the application of an *in vivo* pooled CRISPR screening strategy in chick embryos has uncovered an unexpected role for the super elongation factor component MLLT3 in the differentiation of neural cell fates from caudal epiblast cells. The findings provide new mechanistic insight into the gene regulatory logic underlying neural tube formation and demonstrate the power of this screening platform to interrogate developmental processes at single-cell resolution in an embryonic model system. With further optimization, this approach holds promise for the detailed dissection of gene regulatory mechanisms across diverse tissue contexts.

## MATERIALS AND METHODS

### Egg incubation, embryo staging, electroporation and *ex ovo* culture

Eggs obtained from Henry Stewart & Co. Ltd. were incubated for 36 h at 38°C to generate HH9 embryos with seven somites (Hamburger and Hamilton, 1951). Two types of electroporation were conducted: *in ovo* and *ex ovo* using an Electro Square Porator ECM830. All plasmid mixes were injected via a pulled glass capillary (Harvard Apparatus, EC1 64-0766) and contained SYBR Green (Thermo Fisher Scientific, S7563), PBS, the Cas9 plasmid at a concentration of 500 ng/μl and the pool of guide plasmids at a concentration of 50 ng/µl per individual guide plasmid.

*In ovo* electroporation involved injection of plasmid mix through the head of the HH9 embryo and down the neural tube where electrodes were placed on either side of the neural tube (pulse regime: 30 V, 50 ms length, three pulses, 200 ms interval). The egg was then resealed and placed back into the 38°C incubator for 24 h before collection. *Ex ovo* electroporation followed a protocol similar to that developed by the Stern group (Voiculescu et al., 2008). In short, HH9 embryos were isolated on Whatman filter paper rings, washed twice in Pannett Compton Saline (Table S3) and once in Tyrode's Saline (Table S3), and placed face down in an electroporation chamber with a plate electrode (made in-house by the Crick Making Lab). The plasmid mixture was injected between the vitelline membrane and the embryo over the CLE. The second electrode was placed over the CLE of the embryo to achieve targeted plasmid electroporation (pulse regime: 7 V, 50 ms length, three pulses, 500 ms interval). Embryos were then cultured for 24 h using the EC culture method (Streit, 2008) in 30 mm dishes at 38°C for 24 h before collection.

### Generation of CRISPR and RARA overexpression plasmids

The CRISPR system used was a modified version of that developed in the Sauka-Spengler laboratory (Williams et al., 2018). Both the CAG-Cas9-2A-Citrine (Addgene, #92393) and pcU6_3-MS2-sgRNA (Addgene, #92394) plasmids were kindly donated by the Saurka-Spengler group, expanded, and maxi-prepped using a QIAGEN EndoFree Maxi Plasmid Kit. To modify the pcU6_3-MS2-sgRNA plasmid for capture sequencing, the 10x feature barcoding capture sequence 1 (Table S2) was inserted into hairpin 2 of the stem loop as described on the 10x platform website (Addgene, #221381). To generate the multiple guide plasmids, the stock backbone plasmid was digested with Esp3I (Thermo Fisher Scientific, ER0451), and double-stranded oligos encoding each guide sequence were subsequently ligated into the vector, sequenced, and expanded as described by Williams et al. (2018).

RARα mutant overexpression plasmids (Flajollet et al., 2013) were kindly donated by Philippe Lefebvre (Directeur de Recherche INSERM, Université de Lille - Institut Pasteur de Lille, France) and RARα sequences were PCR amplified with primers adding PaqCI cut sites (New England Biolabs, R0745S), cut via PaqCI and ligated into an EF1alpha-mScarlet backbone (Zhang et al., 2024 preprint). Clones were subsequently sequenced, expanded, and purified using a QIAGEN EndoFree Maxi Plasmid Kit.

### HCR

All HCR followed the manufacturer's protocol (chicken embryos, Multiplexed HCR v.3.0 protocol, Molecular Instruments). In short, HH10 embryos were fixed with 4% paraformaldehyde (PFA) for 1 h and stored in methanol overnight at −20°C. Embryos were then stepwise rehydrated by 25% steps of PBST (0.1% Tween 20 in PBS; Sigma-Aldrich, P2282) on ice, treated with protease K (VWR International, A3830.0500), and fixed again with 4% PFA for an additional 20 min at room temperature. Embryos were then transferred to 5× SSCT (SSC buffer with 0.1% Tween 20; Invitrogen, 15557-044), pre-hybridized for 30 min at 37°C with probe hybridization buffer (Molecular Instruments), incubated for 14 h with 2 pmol of probe, washed four times with 5× SSCT, pre-amplified with amplification buffer (Molecular Instruments) for 5 min at room temperature, incubated for 14 h with hairpin solution in the dark at room temperature, and finally washed four times with 5× SSCT. The hairpins solution was prepared by snap cooling hairpins for 30 min and then mixing to generate a 60 nM solution in amplification buffer. Embryos were then stained with DAPI for 20 min at room temperature, mounted on coverslips with ProLong™ Glass Antifade Mountant (Thermo Fisher Scientific, P36980), and subsequently imaged on a scanning confocal microscope (Leica SP8).

### HCR quantification

HCR images were run through the analysis pipeline described by Rito et al. (2025) to produce a matrix of cell centres, nuclear channel averages, hood channel averages, and cytoplasm channel averages. The hood averages which consist of a slight dilation of the nuclear mask were used as a proxy for total transcript detected per cell. Hood averages were then normalized to DAPI detection to account for imaging artefacts and cells with values larger than the mean of distribution of averages was determined to be positive signal in that channel (Fig. S2B). Hierarchical clustering was performed on cell averages across the imaged channels to produce groups with overlapping HCR detection signatures (Fig. 1G, Fig. S3A,B). From these groups, the threshold of high *TBXT* (notochord) and medium *TBXT* (CLE/PS) was determined to be >0.4 or >0.3, respectively. This was used to quantify the percentage overlap of cells expressing varied levels of *TBXT* and each channel of interest.

### Power analysis quantification

To determine the number of guides per embryo (Fig. S5J), the size of the target tissue was quantified from HCR images of *SOX2* and *TBXT* overlap marking the presumptive CLE (Fig. S5I). This resulted in a total of 7487 cells. We used this to estimate that this would vary from 6000 to 9000 between embryos. The subsequent power analysis for the number of cells that would need to be electroporated with guide to see a significant effect used the following equation:

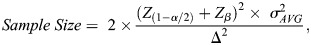
where *α* is the significance level (assigned to 0.05), *β* is the power (80%), and *Z*_*p*_ is the value of a normal distribution that gives the required power and significance (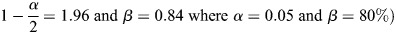. Then Δ is the difference to be detected (50 cell difference in guide detection), *σ* is the standard deviation of guide detection from each target genes, and *σ*_*AVG*_ is the average standard deviation of guide detection across all target genes accounting for unequal populations. The resulting number of cells needed per guide is 240.04, rounded to 240 cells per guide. Considering the size of the tissue ranges from 6000 to 9000 cells and the assumption that electroporation of guides will have about 80% efficacy, this puts the total number of guides that can be electroporated per embryo as 20-30 guides.

### Embryo embedding, sectioning, and immunofluorescence staining

Embryos were either dissected from the eggs or from EC cultures, washed with PBS and fixed for 1 h with 4% PFA (16% stock diluted in PBS; Thermo Fisher Scientific, 28908). Embryos were then washed three times in 0.24 M phosphate buffer solution (PB) (Table S3) and left overnight in 0.12 M PB with 15% sucrose (Sigma-Aldrich, 84100-1KG) at 4°C. Embryos were then embedded in gelatin (Sigma-Aldrich, G2500-500G), frozen in isopentane (between −40°C and −50°C), and stored at −80°C. Blocks were sectioned on a Leica Cryostat CM3050S at 12 μm thickness and placed on Superfrost Plus Adhesion Microscope slides (Epredia, J1800AMNZ).

For immunostaining, slides were washed three times with PBS at 42°C to remove gelatin, then blocked for 1 h in PBS with 5% normal donkey serum (Merck Life Science, D9663) and 0.3% Triton™ X-100 (Sigma-Aldrich, 1002135493), and incubated overnight with primary antibodies at 4°C (Table S4). Slides were then washed four times with PBS, incubated with secondary antibodies (Table S4) for 1-2 h at room temperature, incubated with DAPI for 15 min and washed with PBS. Finally, cover slides were mounted using ProLong™ Glass Antifade Mountant (Thermo Fisher Scientific, P36980), and subsequently imaged on a scanning confocal microscope (Leica SP8). For tortuosity measurements of neural tubes, images were processed in ImageJ to generate masks of the SOX2 HCR channel, marking the neural tube. Then with the ‘Analyze Particles’ function, mask area and perimeter were determined for each neural tube. For the quantification of Pax6^+^ cells, sections were imaged on a scanning confocal microscope (Leica SP8) and nuclei segmented via the analysis pipeline described by Rito et al. (2025) to produce a matrix of cell centres, nuclear channel averages, hood channel averages, and cytoplasm channel averages. Hood averages were then normalized to DAPI detection to account for imaging artefacts and cells with values larger than the mean of distribution of averages was classed as a positive signal in that channel (Fig. S2B). Masks drawn in ImageJ around the neural tube were used to quantify only cells within the neural tube.

### Flow cytometry and FACS

Embryos were isolated and the trunk from the third somite was dissected out and dissociated to a single-cell suspension by incubating in 200 μl of dissociation solution consisting of Accutase (STEMCELL Technologies, 07922) with 3 U/mg papain (Sigma-Aldrich, 10108014001) and 1 mg/ml of collagenase 4 (Gibco, 17104019) for 20 min at 38°C. Additionally, cells were briefly mechanically dissociated after the first 10 min incubation in dissociated solution with a P1000 pipette. Subsequently, 200 μl of DPBS (Thermo Fisher Scientific, 14190144) was added, suspension filtered through a 40 μm Flowmi cell strainer (Sigma-Aldrich, 136800040). Cells were spun for 4 min at 400 ***g*** and resuspended in 0.5% bovine serum albumin (BSA) in PBS (Sigma-Aldrich, A2153) and kept on ice for subsequent analysis.

For the cells intended for single-cell sequencing experiments, Citrine^+^ cells were enriched via FACS using a BD Influx cell sorter with a 70 μm nozzle into DNA Lobind 1.5 ml Eppendorf tubes (Merck, EP0030108051), spun post-sort at 400 ***g*** for 4 min, and resuspended in 50 μl of 0.5% BSA in PBS for subsequent single-cell transcriptomics (described in the ‘scRNA-seq preparation’ section).

For fixed cell flow analysis, single cells dissociated and filtered were spun for 4 min at 400 ***g*** and resuspended in 200 μl DPBS with LIVE/DEAD™ Fixable Dead Stain Near-IR fluorescent reactive dye (Thermo Fisher Scientific, L34976), incubated on ice for 30 min in the dark, spun again for 4 min at 400 ***g*** and finally resuspended in 100 μl of 4% PFA for a 10 min incubation at room temperature. Post-incubation, 1 ml of DPBS was added, and the suspension spun at 2000 ***g*** for 4 min, resuspended in 500 μl of 0.5% BSA in DPBS, and stored at 4°C.

For flow analysis, cell suspensions were resuspended 0.5% BSA and 0.1% Triton X-100 in PBS with primary antibodies (Table S4) and incubated for 2 h at room temperature, washed and then subsequently resuspended in a similar solution with secondary antibodies instead of primary antibodies (Table S4) for 45 min at room temperature. Finally, cells were washed once with 0.5% BSA and 0.1% Triton X-100 in PBS and resuspended in 0.5% BSA in PBS and run on a BD LSRFortessa Cell Analyzer with subsequent analysis in FlowJo where population statistics were calculated by ANOVA and subsequent Tukey tests.

### scRNA-seq preparation

Single-cell suspensions were counted post-FACS and viability of >90% was ensured. A single-cell suspension was loaded onto a Chromium Chip G and run on the 10x Chromium Controller (PN-1000120). The cells were then processed via the Chromium Next GEM Single Cell 3′ Kit v.3.1, 16 rxns (PN-1000268) protocol and the 3′ Feature Barcode Kit, 16 rxns (PN-1000262). cDNA synthesis, library construction, and feature PCR amplification were performed following the manufacturer's protocol. Samples were then sequenced on an Illumina HiSeq4000 using 100 bp paired-end runs aiming for 50,000 reads per cell (between 20,000 and 50,000 depending on the sample).

### Data processing of GEX and CRISPR reads using cell ranger

All GEX datasets were processes using Cell Ranger (v.4.0.0, 10x Genomics); FASTQ files were generated via the Cell Ranger count pipeline and CRISPR barcodes were analysed via the CRISPR guide capture analysis pipeline. FASTQ files were aligned to the white leghorn GRCg7w reference genome with the addition of the Citrine transgene.

(https://www.10xgenomics.com/support/software/cell-ranger/latest/analysis/running-pipelines/cr-feature-bc-analysis)

For the re-analysis of the Rito et al. dataset (Rito et al., 2025) (accession number GSE223189), the 10-somite embryo data (GSM6940809) and the 13-somite embryo data (GSM6940810) were processed in the Cell Ranger count pipeline.

### Seurat analysis of the Rito et al. dataset

From the Cell Ranger pipelines, the Seurat package (version 5) (Butler et al., 2018; Satija et al., 2015) was used in R (v.4.3.3) to analyse the resultant FASTQ files. For the Rito et al. dataset (Rito et al., 2025), removal of outliers for quality control included thresholds based on the numbers of genes/features (nCount_RNA >2500; nFeature_RNA >300 and nFeature_RNA <3500; mitochondrial gene percentage <10% and mitochondrial gene percentage >0.5%). Principal component analysis was used to identify the most variable genes and the top 30 principal components used to define a 16- cluster uniform manifold approximation and projection (UMAP) via the functions ‘FindNeighbors’, ‘FindClusters’, ‘RunUMAP’ and ‘DimPlot’ with a resolution boundary of 0.9. Using known marker genes (*SOX2*, *TBXT*, *PAX6*, *MESP1*, *TBX6*, *NOTO*, *SOX10*, *FOXA2*) the paraxial mesoderm, primitive streak, and neural tube lineages were identified and relevant clusters subsetted into a new dataset using the subset function.

### Feature selection via entropy sorting

As an alternative to highly variable gene selection (HVG) for scRNA-seq feature selection prior to downstream analysis, we used an entropy sorting (Radley et al., 2023) based feature selection algorithm, cESFW (Radley et al., 2024). Using cESFW, we identified a set of 204 genes that facilitated the identification of nine clusters of cells in the primitive streak, pre-neural tube and neural tube of the chick HH10 and HH13 scRNA-seq data (Fig. S1B). We then generated ranked gene lists for each of these nine clusters using the ESS correlation metric. The ESS metric is useful for identifying genes expression of which is specifically enriched in a population interest because it strongly penalizes positive gene expression that is found outside of the group of interest. The nine identified clusters and ranked gene lists were found to be consistent with marker genes highlighted in the literature (Fig. S1A,C). We then took the top 500 cESFW ranked genes in each cluster as candidates for possible genetic regulators of neural fate acquisition (Fig. S1A,B). This identified 2505 differentially expressed genes (Table S1) as some genes overlapped between clusters. The workflow to reproduce these results can be found at the following GitHub repository: https://github.com/aradley/Libby-A-2024.

### Seurat analysis of CRISPR screen

Similar to the Rito et al. dataset (Rito et al., 2025), the screen scRNA-seq dataset was first filtered based on the previously outlined thresholds for genes, features, and mitochondrial DNA percentage; we additionally filtered out a contaminating blood population by removing clusters that expressed high levels of the gene *LMO2*. Then, the screen data was treated as a query dataset and mapped back to the wild-type dataset from Rito et al. using the functions ‘FindTransferAnchors’, ‘TransferData’, ‘AddMetaData’, ‘RunUMAP’ and ‘MapQuery’. This defined a UMAP transformation of the screen dataset to the original wild-type dataset. Cell cycle was scored using the function ‘CellCycleScoring’ and subsequent scores were regressed to normalize the dataset. From here, we similarly subsetted the clusters associated with the paraxial mesoderm, primitive streak, and neural tube lineages and re-clustered following the previously described protocol using highly variable genes to define principal components and obtain a UMAP with 15 defined clusters. We subsequently excluded LMO2^+^ cells (contaminating blood progenitors) leaving 14 total cell populations.

### Gene enrichment analysis

From the protospacer calls determined by the Cell Ranger CRISPR capture sequence pipeline, cell barcodes that had detected guides were identified and added to the metadata of the screen Seurat object. From this, the log10 UMI counts per cell of each protospacer were determined and two standard deviations from the mean log10 UMI count was discarded as ambient amplification. ‘Successful’ guides were determined by using the function ‘FindMarkers’ with the target CRISPR gene as the feature compared between the cells that had received the CRISPR gene of interest guide and those without. From this, fivefold cross-validation testing was used to generate enrichment scores of each individual guide's representations per cluster with guide representation calculated as total cells with guide in cluster divided by the total cells within the cluster. χ^2^ tests comparing the average enrichment across all guides per cluster, scramble 1 enrichment, and the target gene of interest enrichment were used to determine which guides' enrichment patterns deviated from the scramble guide (*z*-score >2 and <−2). This was confirmed by Kullback–Leibler divergence cross-validation tests using the Philentropy R package (*P*<0.05) (Drost, 2018). K-means clustering of the enrichment patterns was determined by minimizing the intra-cluster distances using elbow plot.

### Differential gene expression and GO term analysis of screen

To determine the effect of each gene perturbation beyond reduced expression of the targeted gene the scMAGeCK-LR package (Yang et al., 2020) was used to determine differentially expressed genes post-perturbation compared to the scramble control. Here, the 2502 genes from the ESS ranked gene list were used as the features of the input Seurat object and only genes with *P*<0.05 were considered differentially regulated post-perturbation. The Enrichr package (Chen et al., 2013; Kuleshov et al., 2016; Xie et al., 2021) was then used to identify significant GO terms (GO_Biological_Process_2015) and KEGG Pathways (KEGG_2021_Human) with adjusted *P*-values <0.05.

## Supplementary Material

10.1242/develop.204591_sup1 Supplementary information

Table S1. List of Entropy Sort Score ranked genes
