# Supplementary material for: An in vivo CRISPR screen in chick embryos reveals a role for MLLT3 in specification of neural cells from the caudal epiblast
Source: Development. 2025 Feb 12;152(3):DEV204591. doi: 10.1242/dev.204591 (PMC11883246; doi:10.1242/dev.204591)
Supplement: Supplementary information [file develop-152-204591-s1.pdf]

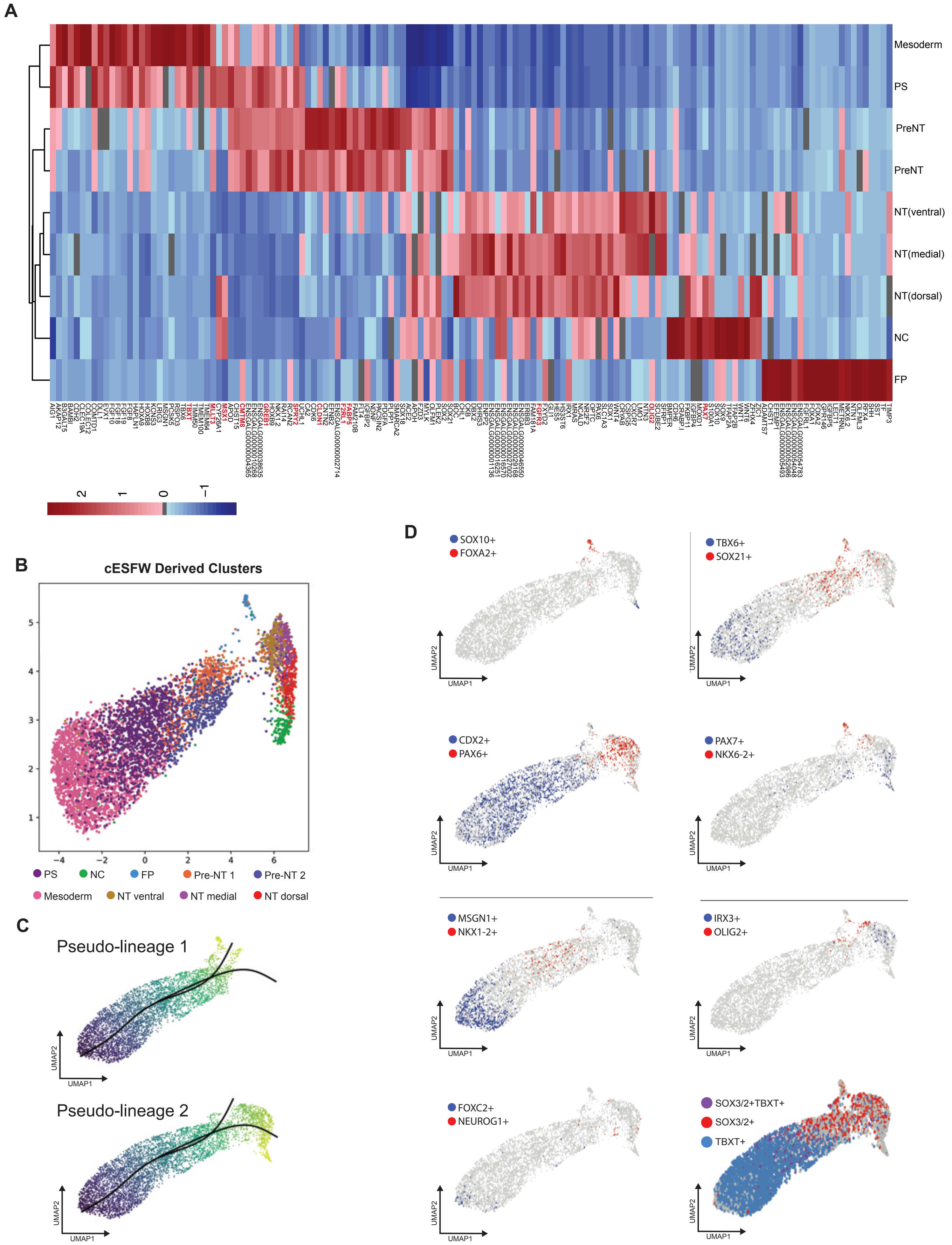
A
Mesoderm
PS
PreNT
PreNT
NT(ventral)
NT(medial)
NT(dorsal)
NC
FP
2
1
0
−1
B
cESFW Derived Clusters
PS
NC
FP
Pre-NT 1
Pre-NT 2
Mesoderm
NT ventral
NT medial
NT dorsal
C
Pseudo-lineage 1
Pseudo-lineage 2
UMAP1
UMAP2
D
SOX10+
FOXA2+
TBX6+
SOX21+
CDX2+
PAX6+
PAX7+
NKX6-2+
MSGN1+
NKX1-2+
IRX3+
OLIG2+
FOXC2+
NEUROG1+
SOX3/2+TBXT+
SOX3/2+
TBXT+

Fig. S1. Establishment of dataset genes of interest and image analysis

A) Heat map showing top 10 ranked Entropy Sort Scored genes across the wildtype dataset where rows refer to pseudo-bulk regions of the reference UMAP in Figure 1B (PS – primitive streak, PreNT – preneural tube, NT – neural tube, NC – neural crest, FP – floor plate). B) UMAP showing cESFW driven clustering of wildtype dataset that was used to define the top 500 genes per cluster. (PS – primitive streak, PreNT – preneural tube, NT – neural tube, NC – neural crest, FP – floor plate) C) Pseudo-lineage trajectories used to generate the heatmap used in Figure 1E. Two trajectories depicted ending in either the neural crest population or the floor plate population. D) UMAPs depicting the gene expression of known lineage marker genes across the wildtype dataset clustered using Louvain clustering based on top ranked Entropy Sorted genes. Bottom right UMAP shows neuromesodermal progenitors identified by the co-expression of *SOX3/2* and *TBXT* expressing cells.

Development • Supplementary information

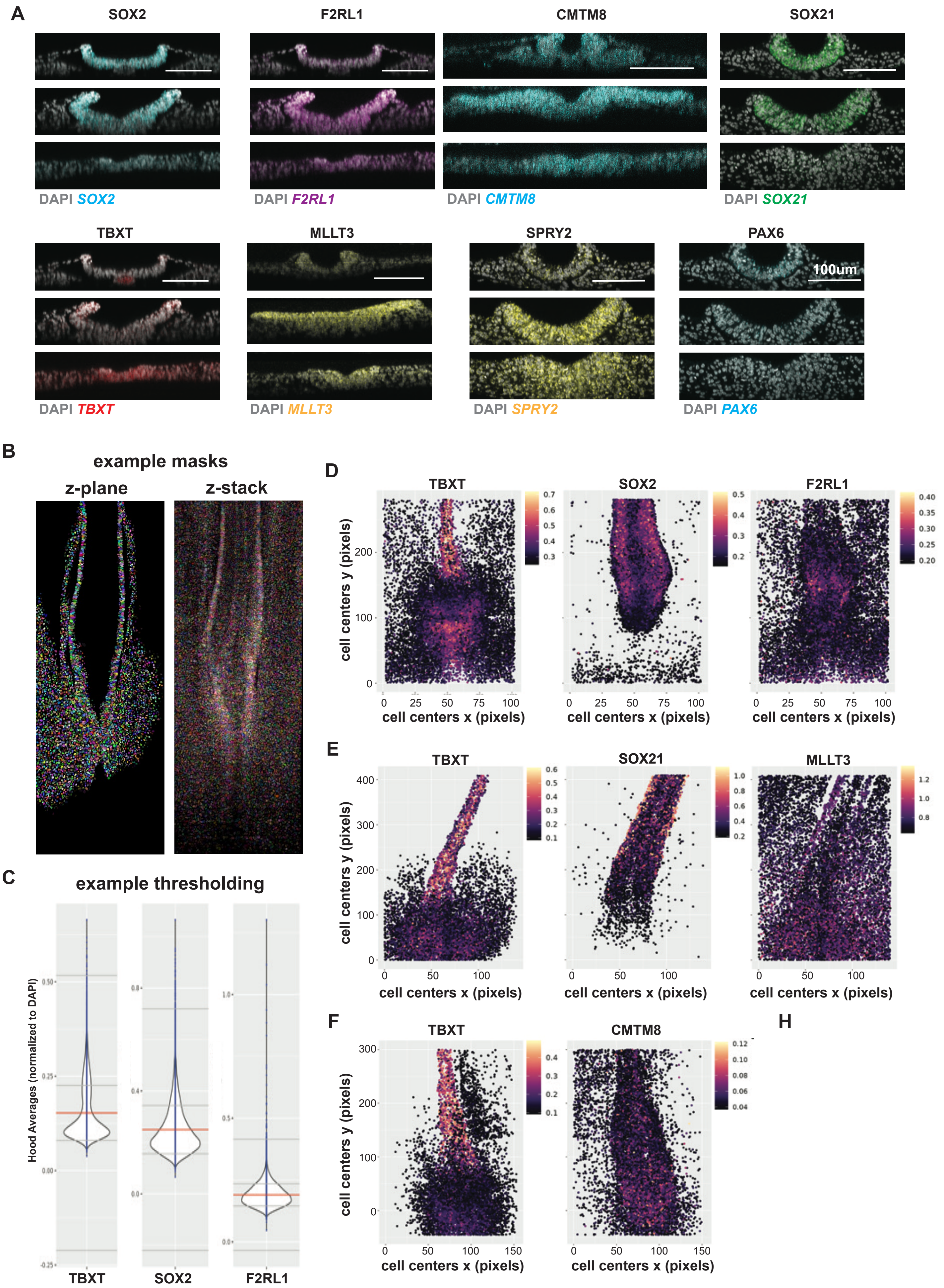
A
SOX2
F2RL1
CMTM8
SOX21
DAPI SOX2
DAPI F2RL1
DAPI CMTM8
DAPI SOX21
TBXT
MLLT3
SPRY2
PAX6
100um
DAPI TBXT
DAPI MLLT3
DAPI SPRY2
DAPI PAX6
B
example masks
z-plane
z-stack
C
example thresholding
Hood Averages (normalized to DAPI)
TBXT
SOX2
F2RL1
D
TBXT
SOX2
F2RL1
cell centers y (pixels)
cell centers x (pixels)
E
TBXT
SOX21
MLLT3
F
TBXT
CMTM8
H

Fig. S2. Quantification of Hybridized Chain Reaction (HCR) images

A) Cross-section images of the HCRs performed in Figure 1F. B) Example image analysis segmentation for quantification of HCR images. C) Example thresholding based on mean values of each channel. Red line indicates the mean. Grey lines indicate one standard deviation away from the mean and 5 standard deviations away from mean. D) Quantification maps from the maximum projected xy plane of *TBXT, SOX2,* and *F2RL1* HCR in HH10 embryos where gene expression detection is normalized to DAPI detection (rostral top-caudal bottom). E) Quantification maps from the maximum projected xy plane of *TBXT, SOX21,* and *MLLT3* HCR in HH10 embryos where gene expression detection is normalized to DAPI detection in the xy plane (rostral top-caudal bottom). F) Quantification maps from the maximum projected xy plane of *TBXT* and *CMTM8* HCR in HH10 embryos where gene expression detection is normalized to DAPI detection (rostral top-caudal bottom).

Development • Supplementary information

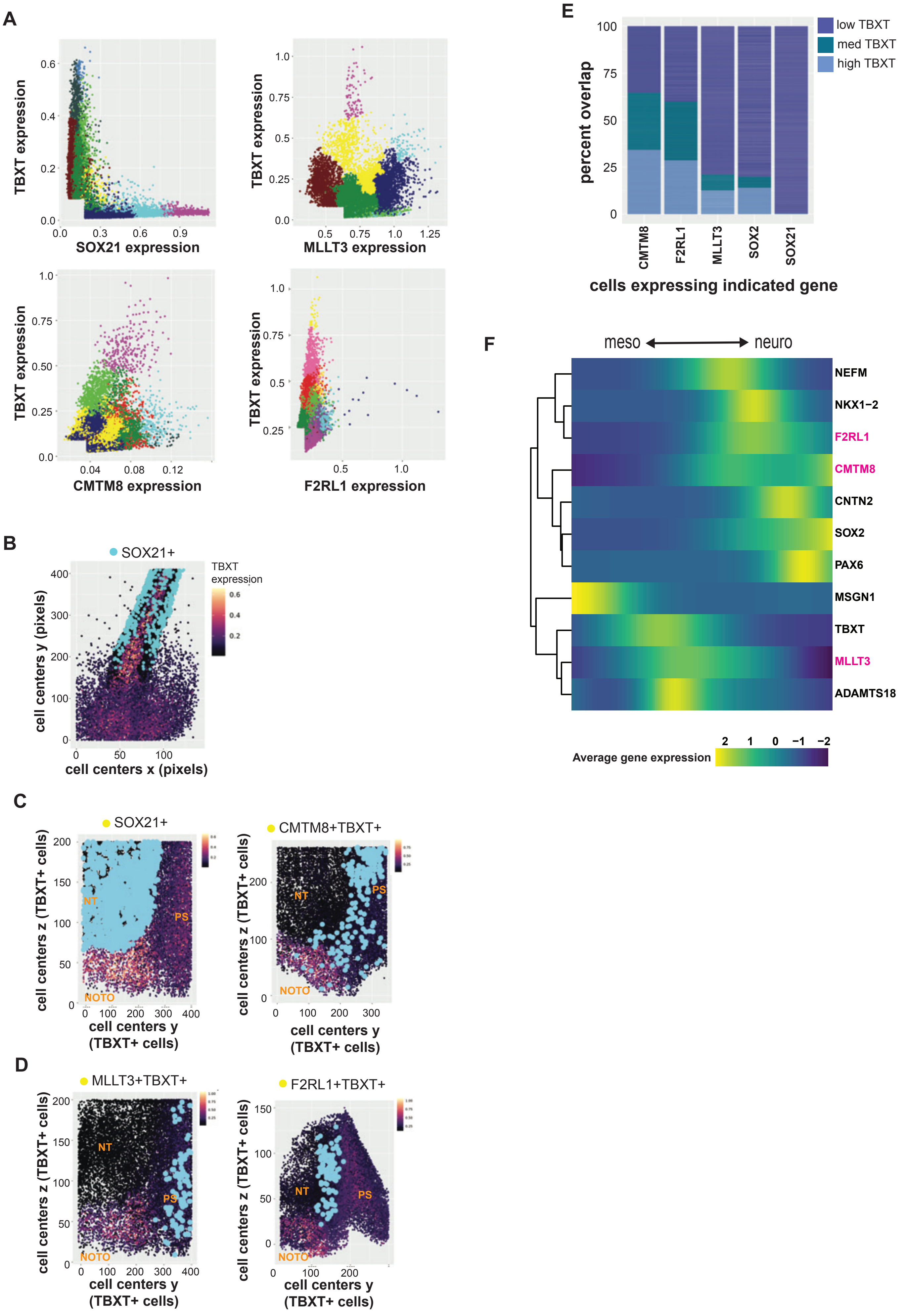

Development • Supplementary information

Fig. S3. Population mapping from HCR quantification to embryo and single cell dataset

A) Hierarchical clustering of *TBXT* transcript count with that of *SOX21, MLLT3, CMTM8*, and *F2RL1*. Light blue dots highlight population plotted in Figure 1I or S3B,C,D. B) Mapping of *SOX21*+ light blue populations from Figure S3A (blue dots) onto *TBXT* expression maps of the imaged embryo in the xy plane (rostral top-caudal bottom). C) Mapping of *SOX21*+ or *CMTM8*+ populations in light blue from Figure S3A onto *TBXT* expression maps of the imaged embryo in the yz plane (rostral-left caudal-right, NT – neural tube, PS – primitive streak, NOTO - notochord). D) Mapping of *MLLT3*+ or *F2RL1*+ populations in light blue from Figure S3A onto *TBXT* expression maps of the imaged embryo in the yz plane (rostral-left caudal-right, NT – neural tube, PS – primitive streak, NOTO - notochord). E) Quantification of cells co-expressing query genes with low, medium, and high levels of *TBXT* expression in quantified HCR images. I) Heatmap depicting pseudo-lineage going from mesodermal (left) to neural (right) of the overlapping expression domains of *MLLT3, F2RL1,* and *CMTM8* with reported markers of CLE and neural tube domains *ADAMTS19, CNTN2, NEFM.* Gene expression in log(10) counts.

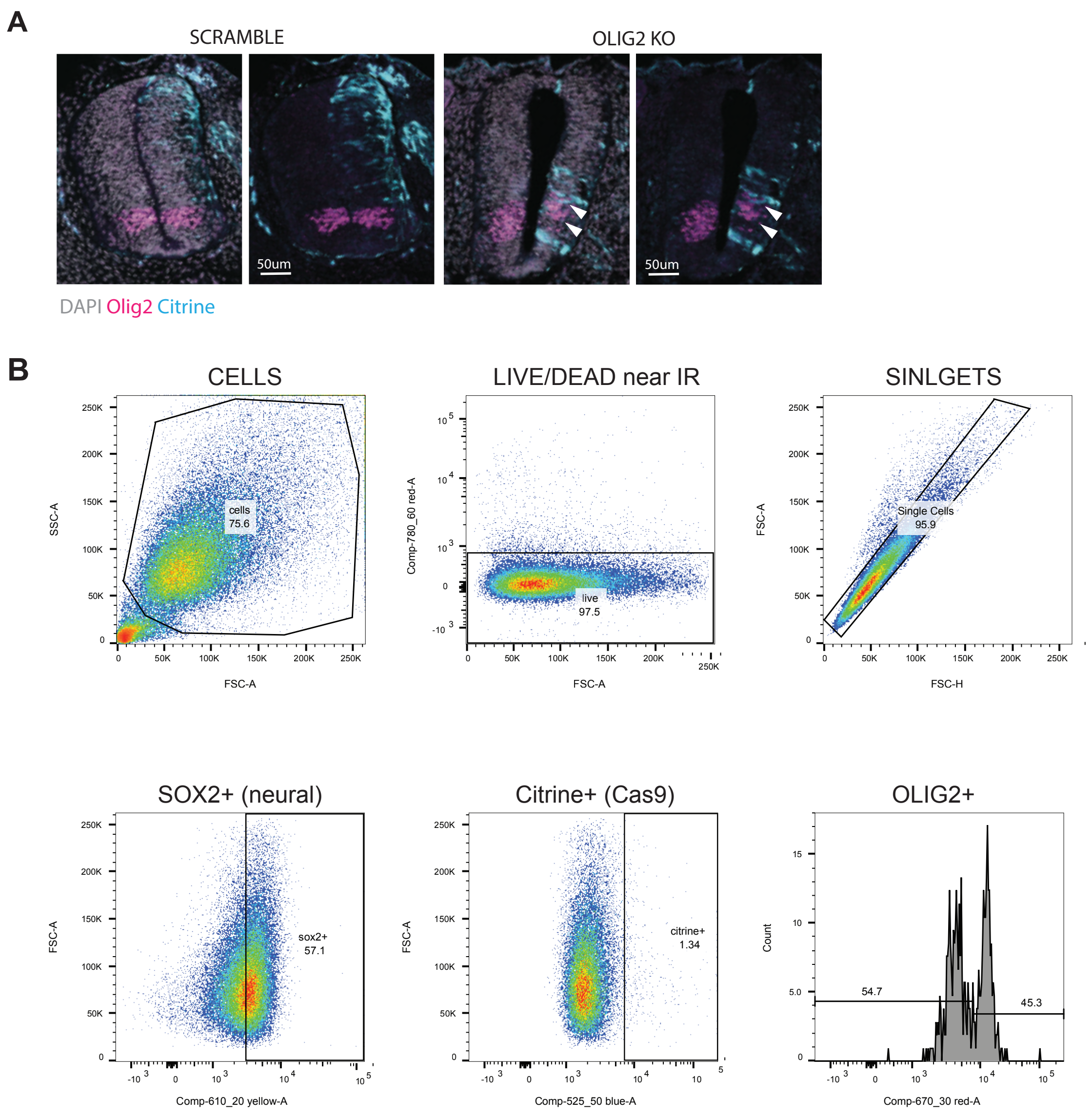

Fig. S4. Flow cytometry of OLIG2 knockout embryos

A) Immunofluorescent images for the indicated makers in transverse sections of the neural tube 24h post electroporation. White arrows mark detection of Cas9 and in KO condition loss of Olig2 protein. B) Example flow gates for quantification of Sox2+ progenitors in embryos and subsequent thresholding for Olig2+ cells.

Development • Supplementary information

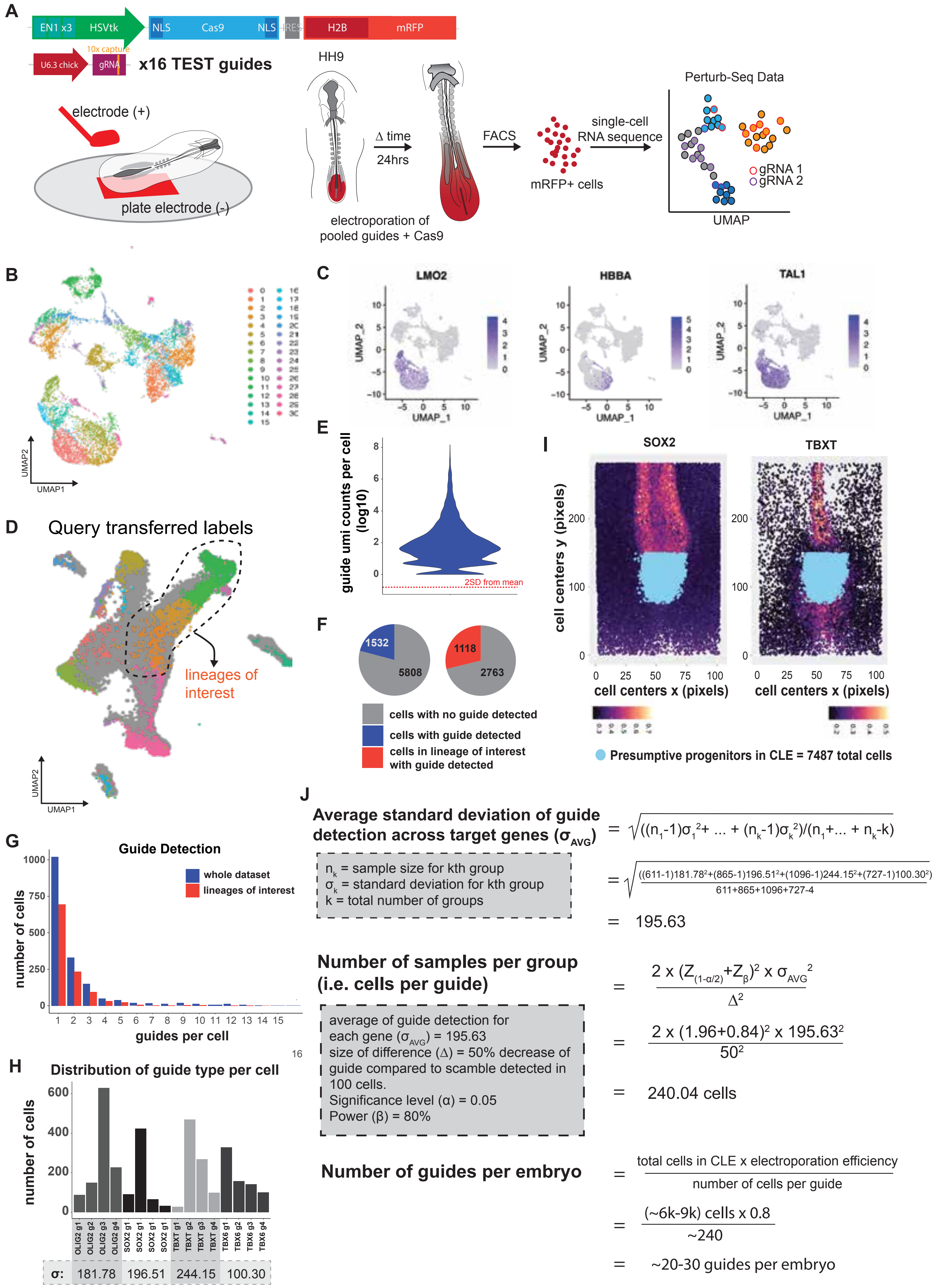
A
EN1 x3
HSVtk
NLS
Cas9
NLS
IRES
H2B
mRFP
10x capture
U6.3 chick
gRNA
x16 TEST guides
electrode (+)
plate electrode (-)
HH9
Δ time
24hrs
electroporation of
pooled guides + Cas9
FACS
mRFP+ cells
single-cell
RNA sequence
Perturb-Seq Data
gRNA 1
gRNA 2
UMAP
B
UMAP2
UMAP1
C
LMO2
HBBA
TAL1
UMAP_2
UMAP_1
D
Query transferred labels
lineages of
interest
UMAP2
UMAP1
E
guide umi counts per cell
(log10)
2SD from mean
F
1532
5808
1118
2763
cells with no guide detected
cells with guide detected
cells in lineage of interest
with guide detected
I
SOX2
TBXT
cell centers y (pixels)
cell centers x (pixels)
Presumptive progenitors in CLE = 7487 total cells
G
Guide Detection
whole dataset
lineages of interest
number of cells
guides per cell
H
Distribution of guide type per cell
number of cells
OLIG2 g1
OLIG2 g2
OLIG2 g3
OLIG2 g4
SOX2 g1
SOX2 g2
SOX2 g3
SOX2 g4
TBXT g1
TBXT g2
TBXT g3
TBXT g4
TBX6 g1
TBX6 g2
TBX6 g3
TBX6 g4
σ:
181.78
196.51
244.15
100.30
J
Average standard deviation of guide detection across target genes (σAVG)
= √((n1-1)σ1²+ ... + (nk-1)σk²)/(n1+... + nk-k)
nk = sample size for kth group
σk = standard deviation for kth group
k = total number of groups
= √(((611-1)181.78²+(865-1)196.51²+(1096-1)244.15²+(727-1)100.30²) / (611+865+1096+727-4))
= 195.63
Number of samples per group
(i.e. cells per guide)
= 2 x (Z(1-α/2)+Zβ)² x σAVG² / Δ²
average of guide detection for
each gene (σAVG) = 195.63
size of difference (Δ) = 50% decrease of
guide compared to scamble detected in
100 cells.
Significance level (α) = 0.05
Power (β) = 80%
= 2 x (1.96+0.84)² x 195.63² / 50²
= 240.04 cells
Number of guides per embryo
= total cells in CLE x electroporation efficiency / number of cells per guide
= (~6k-9k) cells x 0.8 / ~240
= ~20-30 guides per embryo

16

Fig. S5. Preliminary pooled screen in chick embryos

A) Schematic of electroporation and screening workflow. HH8 chicken embryos were electroporated with CRISPR plasmids *ex ovo*, allowed to develop for 24h and subsequently enriched for electroporation via FACS of mRFP positive cells. Cells were profiled by single-cell RNA sequencing. B) UMAP projection of single cell RNA sequencing dataset. C) UMAPs of genes specific to hematopoietic lineage. D) UMAP projection of dataset into wildtype reference map. The dotted line marks the cell types of interest: primitive streak, caudal lateral epiblast (CLE), neural tube. E) Violin plot depicts the range of UMI detection of guides per cell post capture sequence amplification. F) Pie charts depict the proportion of cells with detectable guides across the entire dataset (left, blue) or within the lineages of interest (right, orange). G) Number of guide barcodes detected per cell across dataset (blue) or within lineages of interest (orange). H) Distribution of guides per target detected across the dataset. I) quantification of total cells within the CLE marked by overlap of *TBXT* and *SOX2* expression from HCR images. J) Power analysis for how many guides can be pooled per embryo.

Development • Supplementary information

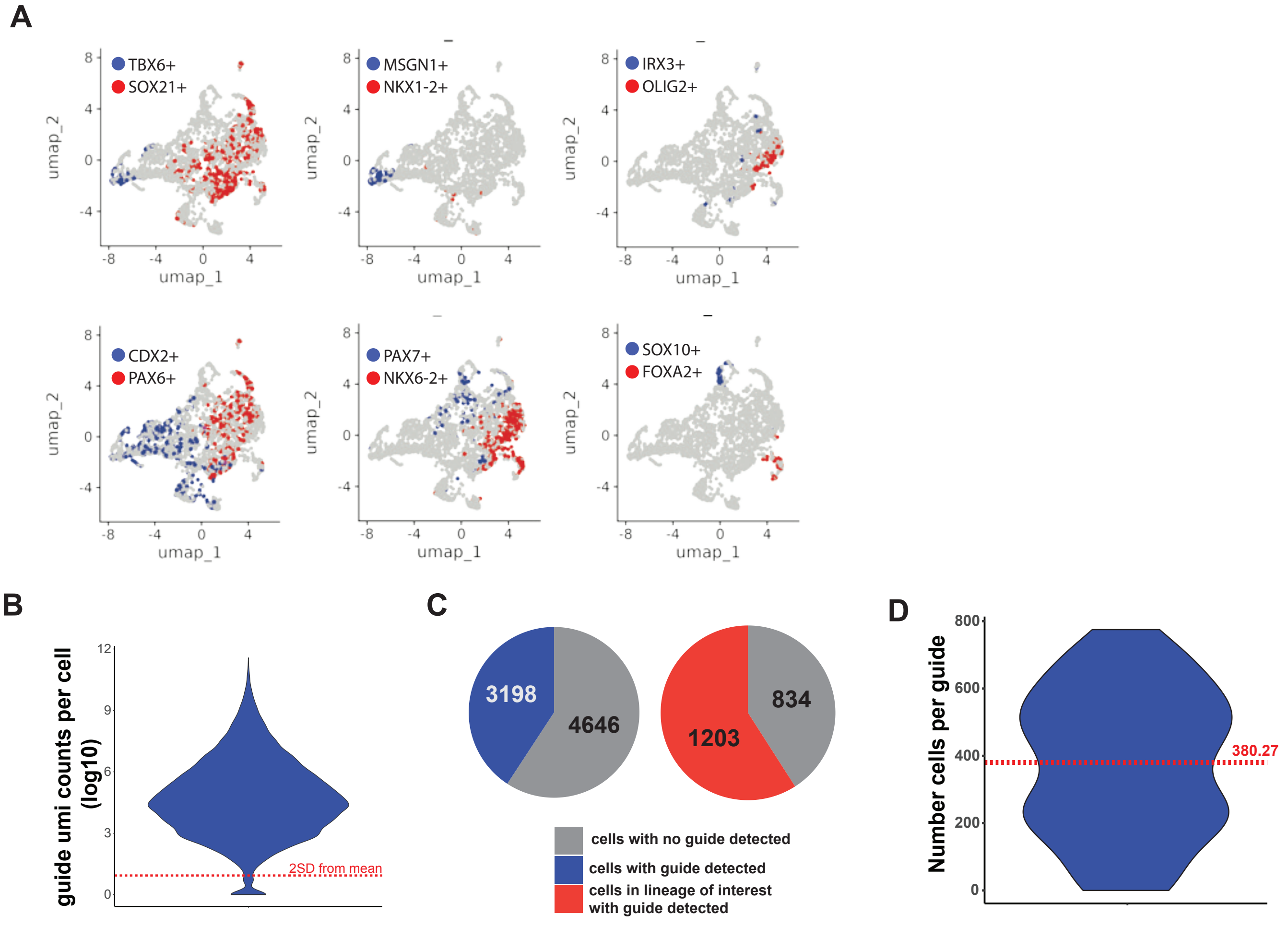

Fig. S6. CRISPR screen dataset analysis and quantification

A) UMAPs depicting the gene expression of known lineage marker genes across the screen RNA sequencing dataset. B) Quantification of the distribution of detected guide UMI counts per cell. Red line marks 2 standard deviations from the mean where guide detection below is considered random RNA amplification. C) Pie charts depict the proportion of cells with detectable gRNAs across the entire dataset (left, blue) or within the lineages of interest (right, orange). D) Distribution of cells per detected guide (red line marks the average number of cells per guide – 380.27).

Development • Supplementary information

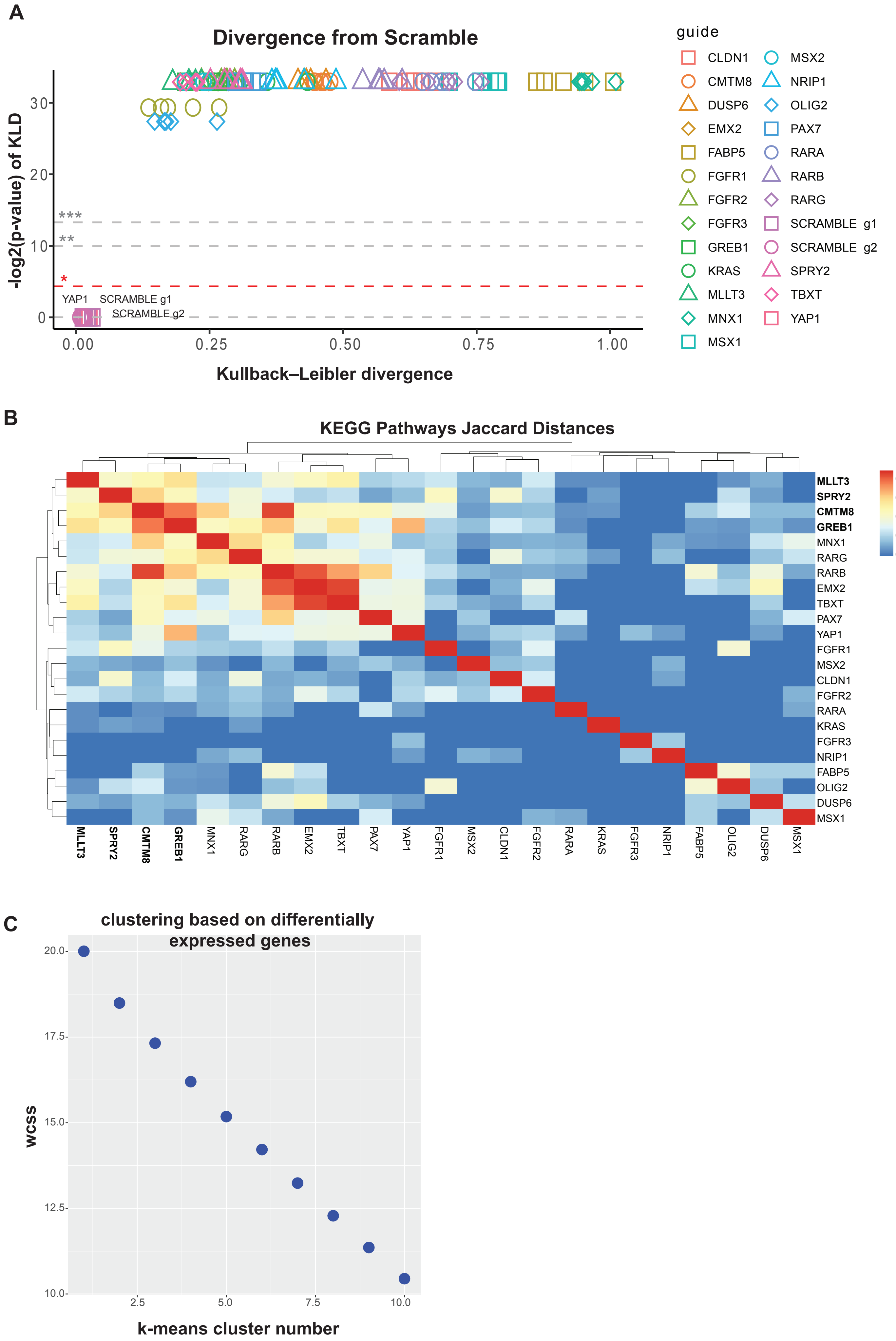

Fig. S7. Analysis of screen guide enrichment and gene expression

A) Quantification of Kullback-Leibler divergence tests of each knockout compared to the distribution of Scramble guide 1. (*,**,*** indicates p = 0.05, p = 0.001, and p = 0.0001, respectively by ANOVA and subsequent Tukey tests) B) Heatmap showing calculated Jaccard distances between gene perturbations based on KEGG pathways called by each knockouts differentially expressed gene list. C) Elbow plot of within cluster sum of square (wcss) as the number of k-means clusters increases when applied to differential gene expression across knockouts.

Development • Supplementary information

Development • Supplementary information

A

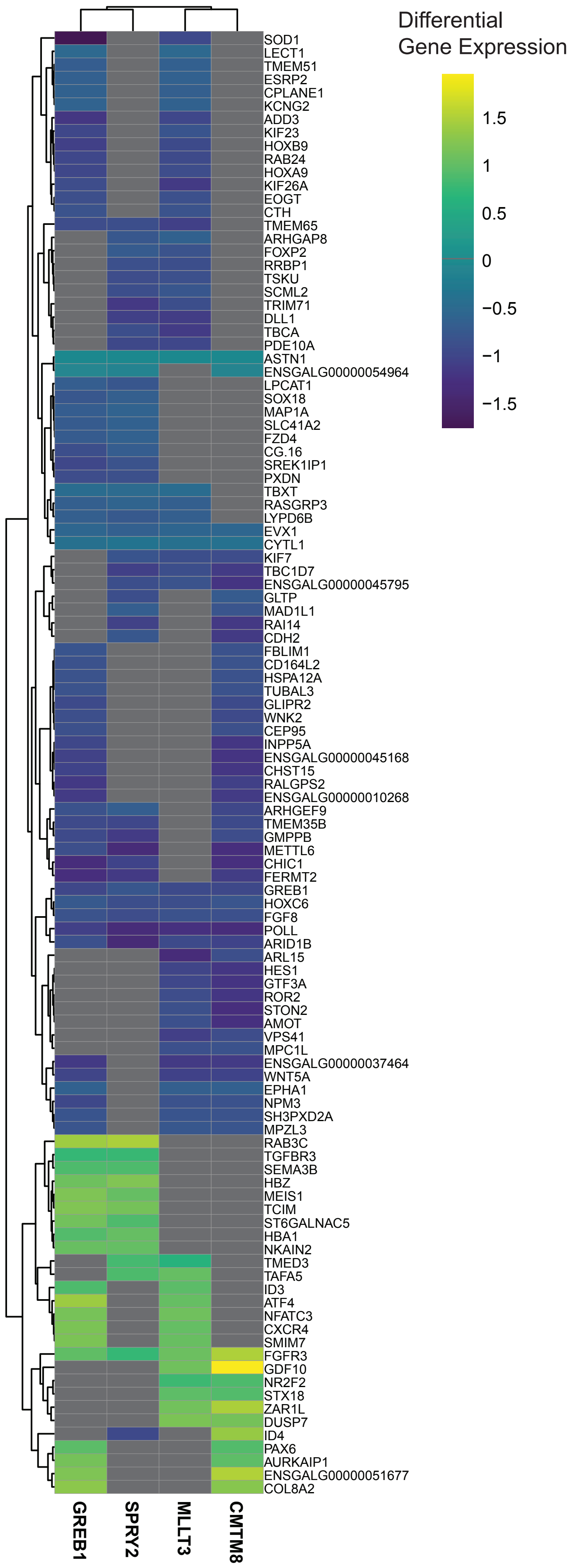

Fig. S8. MLLT3, CMTM8, GREB1, and SPRY2 perturbed cells show overlapping differentially regulated genes

A) Heatmap displaying the overlapping differentially regulated genes between *MLLT3, CMTM8, GREB1*, and *SPRY2* perturbed cells (grey = no differential expression by scMAGeCK algorithm, i.e. p>0.05 by FDR). Gene expression in log(10) counts.

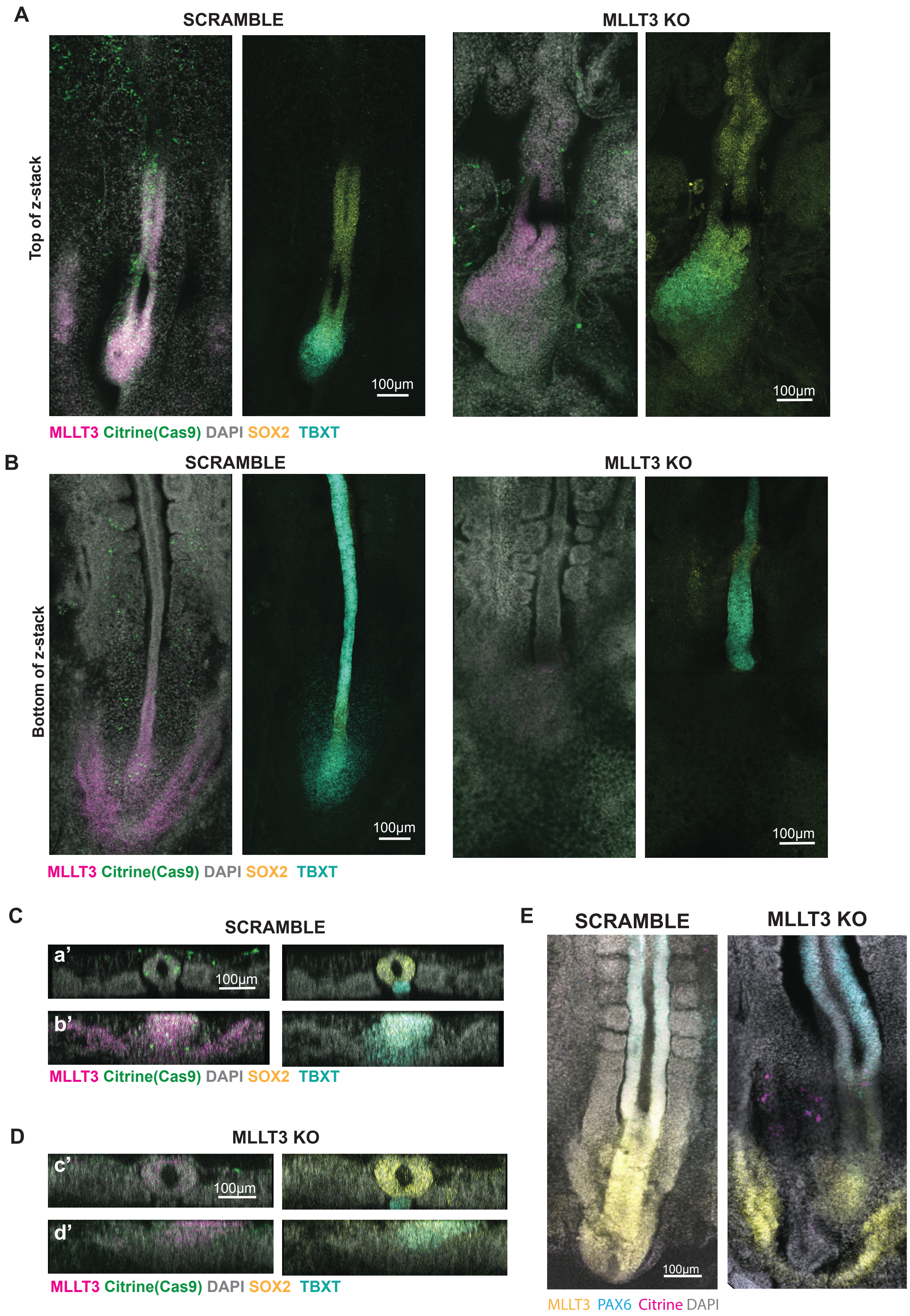

Fig. S9. Quantifications of dual mScarlet and Citrine electroporated embryos A-B) Z-slices of whole mount fluorescence of scramble and *MLLT3* knockout embryos using HCR for *SOX2, TBXT, MLLT3*, and reporter fluorescence of Citrine (marking Cas9 and presumed KO). *(top)* dorsal view of embryo; *(bottom)* ventral view of embryo. C) Optical z-projections of the regions in Figure 6A (a’, b’) depicting scramble control embryos. D) Optical z-projections of the regions in Figure 6A (c’, d’) depicting MLLT3 KO embryos. E) Z-projection of whole mount fluorescence images of scramble ad *MLLT3* knockout embryos using HCR for *PAX6* and *MLLT3*.

Development • Supplementary information

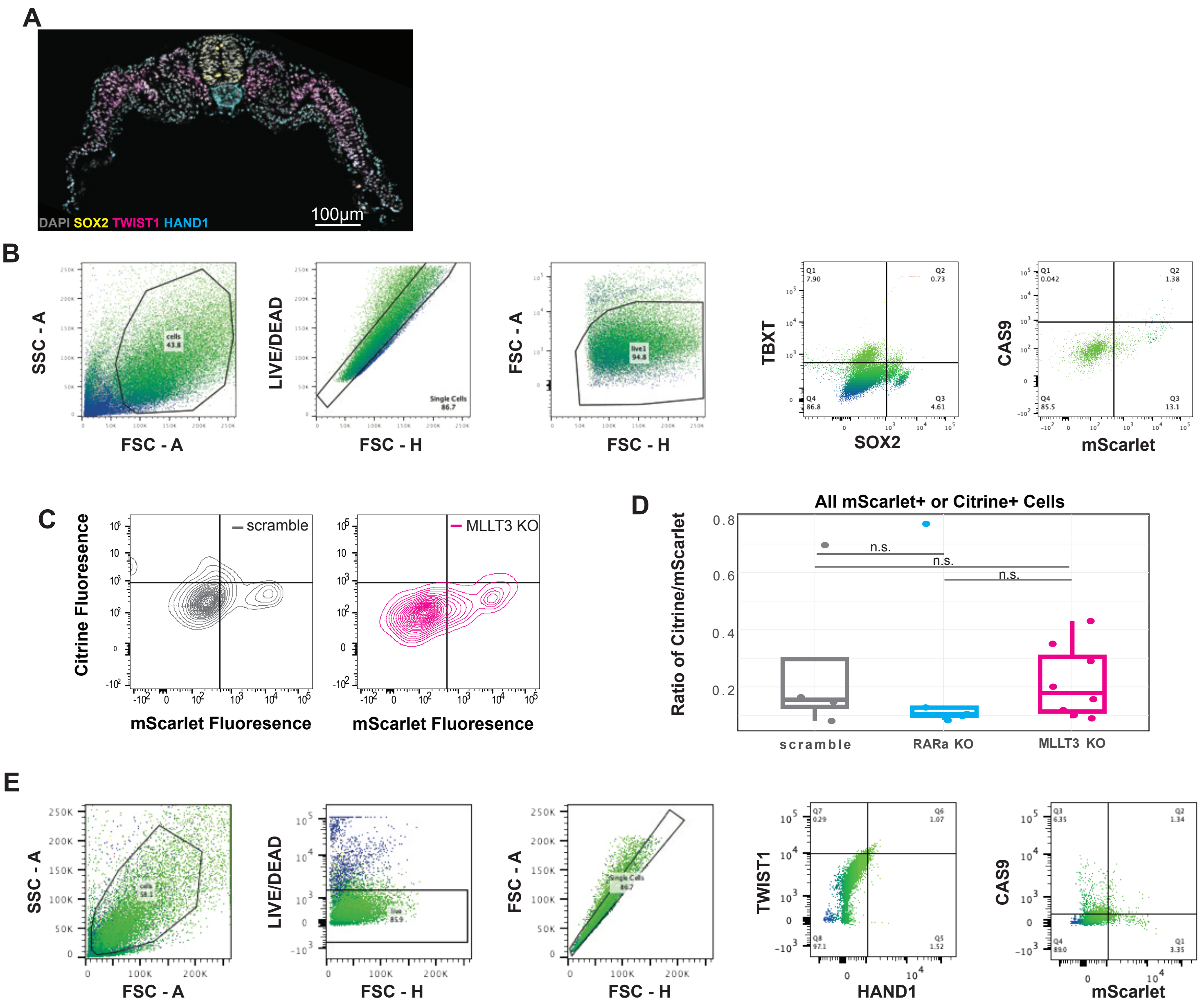

Fig. S10. Quantifications of dual mScarlet and Citrine electroporated embryos

A) Example immunofluorescence image of chick embryo section assayed with antibodies used in flow cytometry experiments. Sox2+ – neural tube, Twist1+Hand1+ – lateral plate mesoderm. B) Flow analysis gates used to for calculation in Figure 6E for Sox2+ neural progenitors. C) Example flow plot of Sox2+ progenitors of either scramble control (grey) or *MLLT3* knockout (pink) where quadrants indicate presence of Citrine or mScarlet populations. D) Quantification of flow cytometry of dually electroporated embryo knockouts showing ratio of Citrine to mScarlet across all cells indicating no significant loss of Citrine cells that have received a targeting guide compared to non-targeting. (n.s. represents non-significance by ANOVA, n = 4-7 embryos per condition). E) Flow analysis gates used to for calculation in Figure 6E for Twist1+Hand1+ lateral plate mesoderm cells.

Table S1. List of Entropy Sort Score ranked genes

Available for download at
https://journals.biologists.com/dev/article-lookup/doi/10.1242/dev.204591#supplementary-data

Development • Supplementary information

Table S2. List of target genes and gRNA sequences

GENE	gRNA#	Capture sequence 1	gRNA seq 5'->3'
**CLDN1**	1	GCTTTAAGGCCGGTCCTAGCAA	CCGCGCAGCCATGGCCAGCG
**CLDN1**	2	GCTTTAAGGCCGGTCCTAGCAA	CAGGAGCTGCAAGCCCCCGC
**CLDN1**	3	GCTTTAAGGCCGGTCCTAGCAA	GGAAGGCCAGCACGAAGCCC
**CLDN1**	4	GCTTTAAGGCCGGTCCTAGCAA	GGCTTCGTGCTGGCCTTCCT
**CMTM8**	1	GCTTTAAGGCCGGTCCTAGCAA	AGATCTTAGGGCTGCTGGTG
**CMTM8**	2	GCTTTAAGGCCGGTCCTAGCAA	GTATTCCGTTCCAGCGATCA
**CMTM8**	3	GCTTTAAGGCCGGTCCTAGCAA	CTGGTGTGGACCCTGATCGC
**CMTM8**	4	GCTTTAAGGCCGGTCCTAGCAA	AGTATTCCGTTCCAGCGATC
**DUSP6**	1	GCTTTAAGGCCGGTCCTAGCAA	GTCTGAACGTATCTAGCATG
**DUSP6**	2	GCTTTAAGGCCGGTCCTAGCAA	ATTTCCGACGCGAAAGGGAC
**DUSP6**	3	GCTTTAAGGCCGGTCCTAGCAA	CATTTCCGACGCGAAAGGGA
**DUSP6**	4	GCTTTAAGGCCGGTCCTAGCAA	TCGCCATTTCCGACGCGAAA
**EMX2**	1	GCTTTAAGGCCGGTCCTAGCAA	CTTGGGCGCGGGCTGAAACA
**EMX2**	2	GCTTTAAGGCCGGTCCTAGCAA	GTGAAACAGCGCTTGGGCGC
**EMX2**	3	GCTTTAAGGCCGGTCCTAGCAA	GGTGAAACAGCGCTTGGGCG
**EMX2**	4	GCTTTAAGGCCGGTCCTAGCAA	TCGATGGTGAAACAGCGCTT
**F2RL1**	1	GCTTTAAGGCCGGTCCTAGCAA	TCAAAAGGAAGAAGTTTTGC
**F2RL1**	2	GCTTTAAGGCCGGTCCTAGCAA	AAGAAGTTTTGCTGGCCAGA
**F2RL1**	3	GCTTTAAGGCCGGTCCTAGCAA	AGATACTAGTAACGCCTCTG
**F2RL1**	4	GCTTTAAGGCCGGTCCTAGCAA	CACTTTGTATGACTCCTCAG
**FABP5**	1	GCTTTAAGGCCGGTCCTAGCAA	TGCCTCTTGCCTTGCAGGTG
**FABP5**	2	GCTTTAAGGCCGGTCCTAGCAA	TTGCCTTGCAGGTGTGGGTA
**FABP5**	3	GCTTTAAGGCCGGTCCTAGCAA	CATGCTTCCCATTTTTCTCA

Development • Supplementary information

**FABP5**	4	GCTTTAAGGCCGGTCCTAGCAA	GGGTATGGCCATGAGAAAAA
**FGFR1**	1	GCTTTAAGGCCGGTCCTAGCAA	ATGTTCGCTTTGGGCAGAGC
**FGFR1**	2	GCTTTAAGGCCGGTCCTAGCAA	TCTGCCCAAAGCGAACATCG
**FGFR1**	3	GCTTTAAGGCCGGTCCTAGCAA	GCCCAAAGCGAACATCGAGG
**FGFR1**	4	GCTTTAAGGCCGGTCCTAGCAA	CATCGAGGTGGAGTCCCACT
**FGFR2**	1	GCTTTAAGGCCGGTCCTAGCAA	GAGATTTGGTATTTGGTTGG
**FGFR2**	2	GCTTTAAGGCCGGTCCTAGCAA	TGAGAGATTTGGTATTTGGT
**FGFR2**	3	GCTTTAAGGCCGGTCCTAGCAA	CGCAACTCAAGTGGTTCTCC
**FGFR2**	4	GCTTTAAGGCCGGTCCTAGCAA	AATTGACAGCGCAACTCAAG
**FGFR3**	1	GCTTTAAGGCCGGTCCTAGCAA	TTCCCTTTGCAGAATACTTG
**FGFR3**	2	GCTTTAAGGCCGGTCCTAGCAA	CACCAACTCTTCCAGAAAGG
**FGFR3**	3	GCTTTAAGGCCGGTCCTAGCAA	AAACACCAACTCTTCCAGAA
**FGFR3**	4	GCTTTAAGGCCGGTCCTAGCAA	GACCGCCTTTCTGGAAGAGT
**GREB1**	1	GCTTTAAGGCCGGTCCTAGCAA	AAGATGGGAAATTCTTATGC
**GREB1**	2	GCTTTAAGGCCGGTCCTAGCAA	CTGGACAGCTCAAGACAACA
**GREB1**	3	GCTTTAAGGCCGGTCCTAGCAA	AGTGTTGCATAATTCTATCG
**GREB1**	4	GCTTTAAGGCCGGTCCTAGCAA	TAAGTTGTTTGAGCGGAGAG
**KRAS**	1	GCTTTAAGGCCGGTCCTAGCAA	AATTACTACTTGCTTTCTGT
**KRAS**	2	GCTTTAAGGCCGGTCCTAGCAA	AGAAAGCAAGTAGTAATTGA
**KRAS**	3	GCTTTAAGGCCGGTCCTAGCAA	GAAAGCAAGTAGTAATTGAT
**KRAS**	4	GCTTTAAGGCCGGTCCTAGCAA	AAAGCAAGTAGTAATTGATG
**MAPK3**	1	GCTTTAAGGCCGGTCCTAGCAA	GTACGCCCCCTCGCCGATGTAGG
**MAPK3**	2	GCTTTAAGGCCGGTCCTAGCAA	GTTGTCCGTGCCGGGAACCGCGG
**MAPK3**	3	GCTTTAAGGCCGGTCCTAGCAA	AACTTTGTTGACGTTATCGTAGG
**MAPK3**	4	GCTTTAAGGCCGGTCCTAGCAA	GCGGGGCCAAGTGTTCGACGTGG

Development • Supplementary information

**MLLT3**	1	GCTTTAAGGCCGGTCCTAGCAA	GTGCGCCGTTCAGGTGAAGC
**MLLT3**	2	GCTTTAAGGCCGGTCCTAGCAA	GTTCAGGTGAAGCTGGAGCT
**MLLT3**	3	GCTTTAAGGCCGGTCCTAGCAA	GGAGCTGGGGCACCGAGCCC
**MLLT3**	4	GCTTTAAGGCCGGTCCTAGCAA	GTTTCTTCCTGACCTGGGCT
**MNX1/Hb9**	1	GCTTTAAGGCCGGTCCTAGCAA	AAATTTTTGGATTTTTCCAT
**MNX1/Hb9**	2	GCTTTAAGGCCGGTCCTAGCAA	GAAATTTTTGGATTTTTCCA
**MNX1/Hb9**	3	GCTTTAAGGCCGGTCCTAGCAA	CAGCCAGCAGCGCGTCGATG
**MNX1/Hb9**	4	GCTTTAAGGCCGGTCCTAGCAA	GCTGGCTGTCGATCCCCCCA
**MSX1**	1	GCTTTAAGGCCGGTCCTAGCAA	GAGGAGAGGAAGAGACAC
**MSX1**	2	GCTTTAAGGCCGGTCCTAGCAA	TCCGCAGCCGGGGCCATGCA
**MSX1**	3	GCTTTAAGGCCGGTCCTAGCAA	GGTGGTCATGTCCGCAGCCG
**MSX1**	4	GCTTTAAGGCCGGTCCTAGCAA	CGGTGGTCATGTCCGCAGCC
**MSX2**	1	GCTTTAAGGCCGGTCCTAGCAA	AAAACCTCCTTCGCTTTGGA
**MSX2**	2	GCTTTAAGGCCGGTCCTAGCAA	GGCTTCTCCTTCCAAAGCGA
**MSX2**	3	GCTTTAAGGCCGGTCCTAGCAA	TTCTCCTTCCAAAGCGAAGG
**MSX2**	4	GCTTTAAGGCCGGTCCTAGCAA	GGAGGTTTTCTCCTCCGACG
**NRIP1**	1	GCTTTAAGGCCGGTCCTAGCAA	CTTTTATTGAACATGACTCA
**NRIP1**	2	GCTTTAAGGCCGGTCCTAGCAA	ATGACTCATGGAGAAGAGCT
**NRIP1**	3	GCTTTAAGGCCGGTCCTAGCAA	GCTTGGCTCTGAGATGCACC
**NRIP1**	4	GCTTTAAGGCCGGTCCTAGCAA	AGGTTAGAACAACAGAATCC
**OLIG2**	1	GCTTTAAGGCCGGTCCTAGCAA	CAGGCTGGCGTCCGAGTCCA
**OLIG2**	2	GCTTTAAGGCCGGTCCTAGCAA	CATGGACTCGGACGCCAGCC
**OLIG2**	3	GCTTTAAGGCCGGTCCTAGCAA	CGGGCGGCTGGAGACCAGGC
**OLIG2**	4	GCTTTAAGGCCGGTCCTAGCAA	AGGACGGGCGGCTGGAGACC
**PAX7**	1	GCTTTAAGGCCGGTCCTAGCAA	AGTGTCCACCCCGCTGGGCC

Development • Supplementary information

**PAX7**	2	GCTTTAAGGCCGGTCCTAGCAA	GGCCCAGCGGGGTGGACACT
**PAX7**	3	GCTTTAAGGCCGGTCCTAGCAA	GCCGGGTGAACCAACTCGGA
**PAX7**	4	GCTTTAAGGCCGGTCCTAGCAA	CAGGGCCGGGTGAACCAACTCGG
**RARA**	1	GCTTTAAGGCCGGTCCTAGCAA	CGCCGCCCTCCCCACAGCCG
**RARA**	2	GCTTTAAGGCCGGTCCTAGCAA	GGGTGACGGGGTAGCCGTTCAGG
**RARA**	3	GCTTTAAGGCCGGTCCTAGCAA	GGCTTGTAGATGCGCGGCAGTGG
**RARA**	4	GCTTTAAGGCCGGTCCTAGCAA	ACATGGTGTACACGTGCCACCGG
**RARB**	1	GCTTTAAGGCCGGTCCTAGCAA	AACTCAGAGCACCAGTTCTG
**RARB**	2	GCTTTAAGGCCGGTCCTAGCAA	TGGAACAAGTTCCTCAGAAC
**RARB**	3	GCTTTAAGGCCGGTCCTAGCAA	CGCGGGGGTGGAAGTGGTGA
**RARB**	4	GCTTTAAGGCCGGTCCTAGCAA	TAAACACGCGGGGGTGGAAG
**RARG**	1	GCTTTAAGGCCGGTCCTAGCAA	CCCAGGGCAACTTACAATGGGGG
**RARG**	2	GCTTTAAGGCCGGTCCTAGCAA	GGCGGGGGTCAATTTGGTCCTGG
**RARG**	3	GCTTTAAGGCCGGTCCTAGCAA	CATAGGAGAACGCAATGCGGGGG
**RARG**	4	GCTTTAAGGCCGGTCCTAGCAA	AGGCGCTGAGGGTTTACGCGCGG
**scramble**	1	GCTTTAAGGCCGGTCCTAGCAA	GCACTGCTACGATCTACACC
**scramble**	2	GCTTTAAGGCCGGTCCTAGCAA	TGCAGTGCTTCAGCCGCT
**SOX2**	1	GCTTTAAGGCCGGTCCTAGCAA	AGUUUGAAUGUACAACAUGA
**SOX2**	2	GCTTTAAGGCCGGTCCTAGCAA	GGGGGCGGGAGGUUUCAGCT
**SOX2**	3	GCTTTAAGGCCGGTCCTAGCAA	CCCCGAAGUUUGCUGGGGGG
**SOX2**	4	GCTTTAAGGCCGGTCCTAGCAA	UCCCCCCGAAGUUUGCUGGG
**SPRY2**	1	GCTTTAAGGCCGGTCCTAGCAA	ATGGAGACGAGAGTTCAGCA
**SPRY2**	2	GCTTTAAGGCCGGTCCTAGCAA	ACGAGAGTTCAGCACGGCAG
**SPRY2**	3	GCTTTAAGGCCGGTCCTAGCAA	CGAGAGTTCAGCACGGCAGC
**SPRY2**	4	GCTTTAAGGCCGGTCCTAGCAA	TCAGCACGGCAGCGGGTCAC

Development • Supplementary information

**TBXT**	1	GCTTTAAGGCCGGTCCTAGCAA	CGCGTCCTCCGGGGAGCCCA
**TBXT**	2	GCTTTAAGGCCGGTCCTAGCAA	GGGGGCGACCATGGGCTCCC
**TBXT**	3	GCTTTAAGGCCGGTCCTAGCAA	GGCGACCATGGGCTCCCCGG
**TBXT**	4	GCTTTAAGGCCGGTCCTAGCAA	CGCCTTGCCCGCGTCCTCCG
**TBX6**	1	GCTTTAAGGCCGGTCCTAGCAA	GGGGAAGCAAGTGCGTCACA
**TBX6**	2	GCTTTAAGGCCGGTCCTAGCAA	CGCACTTGCTTCCCCCAGCC
**TBX6**	3	GCTTTAAGGCCGGTCCTAGCAA	CGGGTAGGGCTCCAGGCTGG
**TBX6**	4	GCTTTAAGGCCGGTCCTAGCAA	GTGATGGAGCTCTGCGGGTA
**YAP1**	1	GCTTTAAGGCCGGTCCTAGCAA	TGACAGGCCAGCACTGACGC
**YAP1**	2	GCTTTAAGGCCGGTCCTAGCAA	AGCACTGACGCAGGGACAGC
**YAP1**	3	GCTTTAAGGCCGGTCCTAGCAA	ACGAACATGCTGAGGGGTCA
**YAP1**	4	GCTTTAAGGCCGGTCCTAGCAA	ATGAGCACGAACATGCTGAG

Development • Supplementary information

Table S3. Solutions

**Solution**	**Components**
Phosphate Buffer (PB) 1M	Mix 684mL of Na_2HPO_4 1M with 316mL of NaH_2PO_4 1M<br><br>Adjust pH to 7.2 with NaH_2PO_4 and Na_2HPO_4
Pannette Compton Saline	SOLN A:<br><br>121g NaCl, 15.5g KCl, 10.42g $CaCl_2$ $2H_2O$ (or 7.7g $CaCl_2$), 12.7g $MgCl_2$ $6H_2O$ to 1000ml dH_2O<br><br>SOLN B:<br><br>2.365g Na_2HPO_4 $2H_2O$, 0.188g NaH_2PO_4 $2H_2O$ to 1000ml dH_2O<br><br>Mix 40ml of Soln A, 900ml dH_2O, and 60ml Soln B
Tyrodes Saline Buffer	10 × concentrated stock: 80 g NaCl, 2 g KCl, 2.71 g $CaCl_2{\cdot}2H_2O$, 0.5 g $NaH_2PO_4{\cdot}2H_2O$, 2 g $MgCl_2{\cdot}6H_2O$, 10 g glucose to 1L of dH_2O

Table S4. Antibodies

Protein Target	Catalog #	Dilution	Citation / RRID
Pax6	Biolegend, 901301	1:200	RRID:AB_2565003
Rfp-Booster	Chromotek, rb2AF568-50	1:1000	RRID:AB_2827576
Gfp-Booster	Chromotek, rb2AF488-50	1:1000	Citation: (*107*)
Pax7	DSHB, AB_528428	1:25	RRID:AB_528428
Sox1	R&D Systems, AF3369	1:250	RRID:AB_2239879
Sox2	Santa Cruz Biotech, sc-365823	1:500	RRID:AB_10842165
Olig2	Milipore , AB9610	1:500	RRID:AB_570666
Olig2	R&D Systems, AF2418	1:500	RRID:AB_2157554
Twist1	Abcam, ab50887	1:300	RRID:AB_883294
Hand1	R&D Systems, AF3168	1:300	RRID:AB_2115853

Development • Supplementary information

AlexaFluor Secondaries	ThermoFisher Scientific	1:1000	RRID:AB_10891079 ,<br>RRID:AB_10894526<br>RRID:AB_10892947<br>RRID:AB_10893739<br>RRID:AB_10892947<br>RRID:AB_10894526<br>RRID:AB_10893040<br>RRID:AB_10893739

Development • Supplementary information
